# Prenatal Tobacco Smoke Exposure Is Associated with Childhood DNA CpG Methylation

**DOI:** 10.1371/journal.pone.0099716

**Published:** 2014-06-25

**Authors:** Carrie V. Breton, Kimberly D. Siegmund, Bonnie R. Joubert, Xinhui Wang, Weiliang Qui, Vincent Carey, Wenche Nystad, Siri E. Håberg, Carole Ober, Dan Nicolae, Kathleen C. Barnes, Fernando Martinez, Andy Liu, Robert Lemanske, Robert Strunk, Scott Weiss, Stephanie London, Frank Gilliland, Benjamin Raby, on behalf of the Asthma BRIDGE consortium

**Affiliations:** 1 Department of Preventive Medicine, Keck School of Medicine, University of Southern California, Los Angeles, California, United States of America; 2 Division of Intramural Research, National Institute of Environmental Health Sciences, National Institutes of Health, Dept of Health and Human Services, Research Triangle Park, North Carolina, United States of America; 3 Channing Division of Network Medicine, Department of Medicine, Brigham and Women’s Hospital, Harvard Medical School, Boston, Massachusetts, United States of America; 4 Norwegian Institute of Public Health, Oslo, Norway; 5 University of Chicago, Chicago, Illinois, United States of America; 6 Johns Hopkins University, Baltimore, Maryland, United States of America; 7 Arizona Respiratory Center, University of Arizona, Arizona, United States of America; 8 National Jewish Health, Denver, Colorado, United States of America; 9 University of Wisconsin, Madison, Wisconsin, United States of America; 10 Washington University School of Medicine, St. Louis, Montana, United States of America; Cleveland Clinic Foundation, United States of America

## Abstract

**Background:**

Smoking while pregnant is associated with a myriad of negative health outcomes in the child. Some of the detrimental effects may be due to epigenetic modifications, although few studies have investigated this hypothesis in detail.

**Objectives:**

To characterize site-specific epigenetic modifications conferred by prenatal smoking exposure within asthmatic children.

**Methods:**

Using Illumina HumanMethylation27 microarrays, we estimated the degree of methylation at 27,578 distinct DNA sequences located primarily in gene promoters using whole blood DNA samples from the Childhood Asthma Management Program (CAMP) subset of Asthma BRIDGE childhood asthmatics (n = 527) ages 5–12 with prenatal smoking exposure data available. Using beta-regression, we screened loci for differential methylation related to prenatal smoke exposure, adjusting for gender, age and clinical site, and accounting for multiple comparisons by FDR.

**Results:**

Of 27,578 loci evaluated, 22,131 (80%) passed quality control assessment and were analyzed. Sixty-five children (12%) had a history of prenatal smoke exposure. At an FDR of 0.05, we identified 19 CpG loci significantly associated with prenatal smoke, of which two replicated in two independent populations. Exposure was associated with a 2% increase in mean CpG methylation in *FRMD4A* (p = 0.01) and Cllorf52 (p = 0.001) compared to no exposure. Four additional genes, *XPNPEP1*, *PPEF2*, *SMPD3* and *CRYGN*, were nominally associated in at least one replication group.

**Conclusions:**

These data suggest that prenatal exposure to tobacco smoke is associated with reproducible epigenetic changes that persist well into childhood. However, the biological significance of these altered loci remains unknown.

## Introduction

Smoking while pregnant is associated with a myriad of negative health outcomes both for the mother and for the fetus. [1] *In utero* tobacco smoke exposure (IUS) can damage the placental structure and function [2], is associated with changes in children’s neurodevelopment and behavior [3] as well as with impaired lung function and increased risk of developing asthma. [4], [5], [6] Moreover, IUS-related deficits in lung function are larger for children with asthma. [7].

One hypothesized mechanism through which IUS may act is by altering the epigenetic landscape within the developing fetus. Increasingly, scientific reports are linking IUS exposure to alterations in the fetal epigenome, including changes in DNA methylation in numerous genes and tissue types. [8], [9] To further investigate the association between IUS and epigenetics, we evaluated the association between IUS exposure and DNA methylation in the first phase of subjects participating in the Asthma BioRepository for Integrative Genomic Exploration (Asthma BRIDGE) study - a subset of asthmatic children originally from the Childhood Asthma Management Program (CAMP) trial. [10], [11] We designed a study aimed at investigating DNA methylation in promoter regions of genes as these are the most pertinent regulatory regions for gene expression. We interrogated 27,578 CpG loci using the Illumina HumanMethylation27 platform - a promoter-centric assay - measured in whole blood samples collected in the CAMP population and replicated our findings in two other populations: 1) the remainder of the Asthma BRIDGE project with Illumina HumanMethylation450 (HM450) data; and 2) the Norwegian Mother and Child Cohort Study (MoBa).

## Methods

### Study Population

Asthma BRIDGE is a multicenter initiative to develop a publicly accessible resource consisting of ∼1,500 asthmatics and controls with comprehensive phenotype and genomic data. The first phase of the Asthma BRIDGE multicenter initiative consisted of asthmatic subjects that were originally recruited as part of CAMP, the details of which have been described elsewhere. [10], [11] Briefly, CAMP is a multicenter, randomized, double-masked clinical trial to compare the long-term effectiveness and safety of 3 inhaled treatments for asthma: budesonide, nedocromil, and placebo. Asthma was defined by the presence of asthma symptoms or the use of an inhaled bronchodilator at least twice per week or use of a daily asthma medication for the 6 months before the screening interview. All participants had increased airway responsiveness to methacholine (PC20<12.5 mg/mL) at study entry. Each parent or guardian signed a consent form and each participant 7 years of age and older signed an assent form approved by each clinical center’s institutional review board. Prior to treatment randomization, DNA samples were collected from 968 of 1,041 willing CAMP participants, of which 572 were of self-reported Western European ancestry. Of these, sufficient DNA for methylation typing was available for 554 (97%). Mean age at DNA collection was 9 years.

Replication was conducted in the remainder of Asthma BRIDGE subjects and in MoBA.

Asthma BRIDGE had 526 participants for whom whole blood samples with DNA methylation from the Illumina HM450 were available, of whom 332 were asthmatic. Subjects were recruited from five studies: Genomic Research on Asthma in the African Diaspora (GRAAD), Children’s Health Study (CHS), Chicago Asthma Genetics (CAG), Childhood Asthma Research and Education (CARE), and Mexico City Childhood Asthma Study (MCCAS). Descriptions of these cohorts are provided in File S1. The primary objective of the Asthma BRIDGE initiative was to create a biorepository of cell lines and accompanying datasets for public access. All of the primary data analyzed for this study have been submitted to BioLINCC (https://biolincc.nhlbi.nih.gov), from where it will be made available to the public. The data is also submitted to dbGaP (http://www.ncbi.nlm.nih.gov/gap) within 6 months of publication.

The Norwegian Mother and Child Cohort Study (MoBa) consisted of 1062 participants in whom the association between maternal cotinine level and DNA methylation in cord blood was recently assessed using Illumina’s Infinium HumanMethylation450 (HM450) BeadChip. [8] Maternal plasma cotinine concentrations in the MoBa samples were measured using liquid chromatography–tandem mass spectrometry. [12].

### Ethics Statement

Written informed consent was provided by all study subjects. The institutional review boards (IRB) of the Brigham and Women’s Hospital, and each of the participating CAMP study centers, approved these protocols. The MoBa study was approved Norwegian Data Inspectorate and the Regional Ethics Committee for Medical Research and the NIEHS Institutional Review Board.

### Maternal Smoking Exposure

Information regarding IUS exposure was obtained during the baseline medical interview for CAMP and Asthma BRIDGE subjects. Children were considered to have IUS exposure if respondents answered affirmatively to the question, ‘‘Did this child’s mother smoke while she was pregnant with this child?’’ Information about current environmental tobacco smoke exposure was obtained during follow-up interviews with the question, ‘‘Do any caretakers of the child currently smoke cigarettes?’’ In MoBa, cotinine concentrations were measured in maternal samples collected at about 18 weeks of pregnancy using liquid chromatography–tandem mass spectrometry. [12], [13]

### DNA Methylation

Laboratory personnel performing DNA methylation analysis were blinded to study subject information. DNA was extracted whole blood cells using the QiaAmp DNA blood kit (Qiagen Inc, Valencia, CA) and stored at −80 degrees Celcius. Two micrograms of genomic DNA from each sample were treated with bisulfite using the EZ-96 DNA Methylation Kit (Zymo Research, Irvine, CA, USA), according to the manufacturer’s recommended protocol and eluted in 18 ul. The results of the HM27 assay were compiled for each locus as previously described and were reported as beta (β) values. [14] Details of quality control procedures, dilution control series, and calculation of β values are provided in File S1.

CpG loci on the HM27 array were removed from analyses if they were on the X and Y chromosomes, or if they contained SNPs, deletions, repeats, or if they mapped to multiple places in the genome. A Normal exponential background correction was first applied to the raw intensities at the array level to reduce background noise. [15] We then normalized each sample’s methylation values to have the same quantiles to address sample to sample variability. [16] Lastly, we applied COMBAT, a mixed effects model that accounted for exposure, to reduce probe level variation. [17] The density plots after each step are shown in Figure S1 (in File S1), and illustrate the improvement acquired from each correction applied.

Samples from 554 CAMP participants were included for initial analysis of DNA methylation. Six samples were excluded for having methylation call rates <95% and an additional 17 were excluded because their sample mean methylation across all probes was greater than 2 standard deviations from the overall mean of all samples on the plate. Two additional samples were removed for discrepancy of sex determination when comparing questionnaire response data to X chromosome methylation patterns. One additional sample was removed for missing IUS exposure and another was removed before COMBAT correction for being the only sample on a single chip. This left 527 samples for analysis.

### mRNA Expression

Paired samples of isolated mRNA and DNA collected at the same point in time were available for the Asthma BRIDGE replication dataset only. We collected 5 cc of whole blood in RNA PaxGene tubes. Samples were shipped to the data coordinating center monthly for RNA extraction using the PAXgene Blood RNA Kit according to manufacturer’s protocol (PreAnalytix). Expression profiles were generated using the Illumina Human HT-12 v4 arrays, according to manufacturers’ protocol (Illumina, San Diego CA). Preprocessing was performed using quantile normalization with the *lumi* Bioconductor package. [18] Evidence for association between CpG methylation and target gene expression was assessed using linear regression, including adjustment for age, gender, and clinical center.

### Statistical Analyses

Descriptive analyses were performed to examine the distribution of subject characteristics. Density plots of DNA methylation values were created and evaluated for quality control. Outlier DNA methylation values were identified as values that were either greater than the median+3*IQR or less than the median- 3*IQR and were removed from analyses.

To investigate the association between IUS exposure and percent DNA methylation, we fitted beta regression models adjusted for age, sex, clinic and cell type. The following cell types were estimated using the method of Houseman et al [19]: B-lymphocytes, granulocytes, monocytes, natural killer cells, CD4+ T-lymphocytes, and CD8+ T-lymphocytes. Beta regression was used to address the non-normal distribution of DNA methylation values, which are bounded by 0 and 1 and in many cases heavily skewed toward one end or the other. [20] The beta regression model is as follows: Let y_1_,…,y_n_ be a random sample of DNA methylation beta values for a single feature on the HM27 assay. We assume y_i_∼Beta(µ_i_,φ), i = 1,…,n, with E(y) = μ and Var(y) = μ(1-μ)/(1+φ), the parameter φ measuring precision. The regression model is defined as logit(µ_i_) = a+a_1_ X_i1_+ …+ a_k_ X_ik_, with X_1_ measuring IUS exposure and X_2_,…,X_k_ measuring age, sex, clinic, and estimated cell type fractions for B-lymphocytes, granulocytes, monocytes, natural killer cells, CD4+ T-lymphocytes, and CD8+ T-lymphocytes. The precision parameter is assumed constant for all observations. All regressions were run in R using the betareg package (version R2.15.3).

Beta regression requires the data to be between zero and one, therefore a shrinkage method was applied to force the zeros to be positive as follows: 0.999999*(meth value-0.5) +0.5. Because beta regression is modeled on the logit scale, we also present estimates from linear regression models to provide interpretation of a % difference in methylation and compare effect estimates to those from our replication population. All regression analyses were adjusted for multiple testing at a false discovery rate (FDR) of 0.05, using the method of Benjamini and Hochberg. [21]

To investigation the association between methylation or IUS and expression, linear regression models were fit in which expression values were transformed on the log2 scale. Sensitivity analyses were conducted in regression models of our top 19 loci to evaluate potential confounding by the following covariates: income, education, parental history of asthma, asthma severity, maternal or paternal smoking in childhood. To replicate results, we ran identical models in the 526 participants in Asthma BRIDGE, as well as within asthmatics only. We also compared our results to those from a study of 1062 participants from the Norwegian Mother and Child Cohort Study. [8] Joubert et al examined the association between maternal plasma cotinine (active smoking defined as >56.8 nmol/L) and cytosine methylation in cord blood using robust linear regression on the log-ratio of the methylation beta value obtained from the Illumina HM450 array, adjusting for several covariates.

All tests assumed a two-sided alternative hypothesis, a 0.05 significance level, and were conducted using the R programming language, version R2.15.3.

## Results

The HM27 assay demonstrated high reproducibility, with an average spearman correlation coefficient of 0.96 across all replicate control samples (i.e. PBL and dilution controls). Additional analyses of accuracy, assessed by calculating bias within the dilution controls, suggested minimal bias, with values of 0.15, 0.07, −0.03, and −0.11 for 10%, 35%, 60% and 85% dilution controls, respectively.

Of the 527 children in the study, 65 (12.3%) were exposed to maternal smoking during pregnancy. Age and sex did not vary between exposed and unexposed children. However, unexposed children were more likely to have a maternal (26%) or paternal (21%) history of asthma compared to exposed children (16%). Mothers who smoked during pregnancy also had significantly lower education and income levels than mothers who did not smoke (Table 1).

**Table 1:** Descriptive characteristics of the CAMP study population (n = 527).

				*Exposure to maternal smoking in utero*				
		Overall		No (N = 462)		Yes (N = 65)		
Characteristics	level	Count	%	Count	%	Count	%	p-values*
*In utero* tobacco smoke exposure		65	12.3					
Male		320	60.7	280	60.6	40	61.5	0.89
Maternal history of asthma		129	25.0	119	26.2	10	16.1	0.09
Paternal history of asthma		103	20.6	94	21.3	9	15.8	0.34
Maternal education	≤ high school	87	16.5	60	13.0	27	41.5	<0.0001
	some college	203	38.6	175	38.0	28	43.1	
	college degree	236	44.9	226	49.0	10	15.4	
Clinic site	Albuquerque	45	8.5	37	8.0	8	12.3	0.15
	Baltimore	79	15.0	64	13.9	15	23.1	
	Boston	41	7.8	36	7.8	5	7.7	
	Denver	68	12.9	61	13.2	7	10.8	
	San Diego	53	10.1	50	10.8	3	4.6	
	Seattle	86	16.3	80	17.3	6	9.2	
	St. Louis	91	17.3	81	17.5	10	15.4	
	Toronto	64	12.1	53	11.5	11	16.9	
Age (mean(SD))		8.7	2.1	8.7	2.1	8.7	2.1	0.92

*p-values are calculated using chi-sq test.

After application of background correction and normalization procedures, we observed a total of 26 loci that were statistically significantly associated with IUS at a FDR <0.05. However, 7 of these loci were removed from further consideration because their mean methylation levels were less than 3% or greater than 97% and thus largely invariant. The remaining 19 loci are shown in Table 2.

**Table 2:** CpG Loci significantly associated with IUS exposure in CAMP asthmatics (N = 527).

Probe ID	Symbol	Chr	Mean methylation level	β*	Difference in methylation**	FDR p-value
cg05697249	C11orf52	11	0.66	0.09	0.02	0.001
cg14724265	PPEF2	4	0.76	0.17	0.03	0.01
cg25464840	FRMD4A	10	0.75	0.13	0.02	0.01
cg20588045	PCDH15	10	0.28	0.10	0.02	0.01
cg20555507	TRPM3	9	0.08	0.13	0.01	0.03
cg16184943	ZNF280B	22	0.34	−0.09	−0.02	0.03
cg09352789	XPNPEP1	10	0.29	−0.06	−0.01	0.03
cg07499072	FST	5	0.04	0.11	0.004	0.03
cg22830895	CRYGN	7	0.43	0.11	0.03	0.03
cg13473383	ZDHHC5	11	0.09	−0.08	−0.01	0.03
cg10556064	SMPD3	16	0.58	0.10	0.02	0.03
cg04112019	IGF2AS	11	0.12	0.07	0.01	0.03
cg00169548	BAZ1A	14	0.48	−0.10	−0.02	0.03
cg10493739	TMEM38B	9	0.06	−0.07	−0.004	0.03
cg01058368	CDH10	5	0.82	0.12	0.02	0.04
cg09143663	BACH1	21	0.72	0.08	0.02	0.04
cg20773127	ENPEP	4	0.58	0.10	0.02	0.05
cg24956866	CALD1	7	0.58	0.10	0.02	0.05
cg14580737	RFXANK	19	0.80	−0.10	−0.02	0.05

* coefficient from beta regression adjusted for age, sex, and clinic and cell type.

**coefficient from linear regression model illustrating difference in methylation level comparing IUS exposed to unexposed.

**Figure 1:**
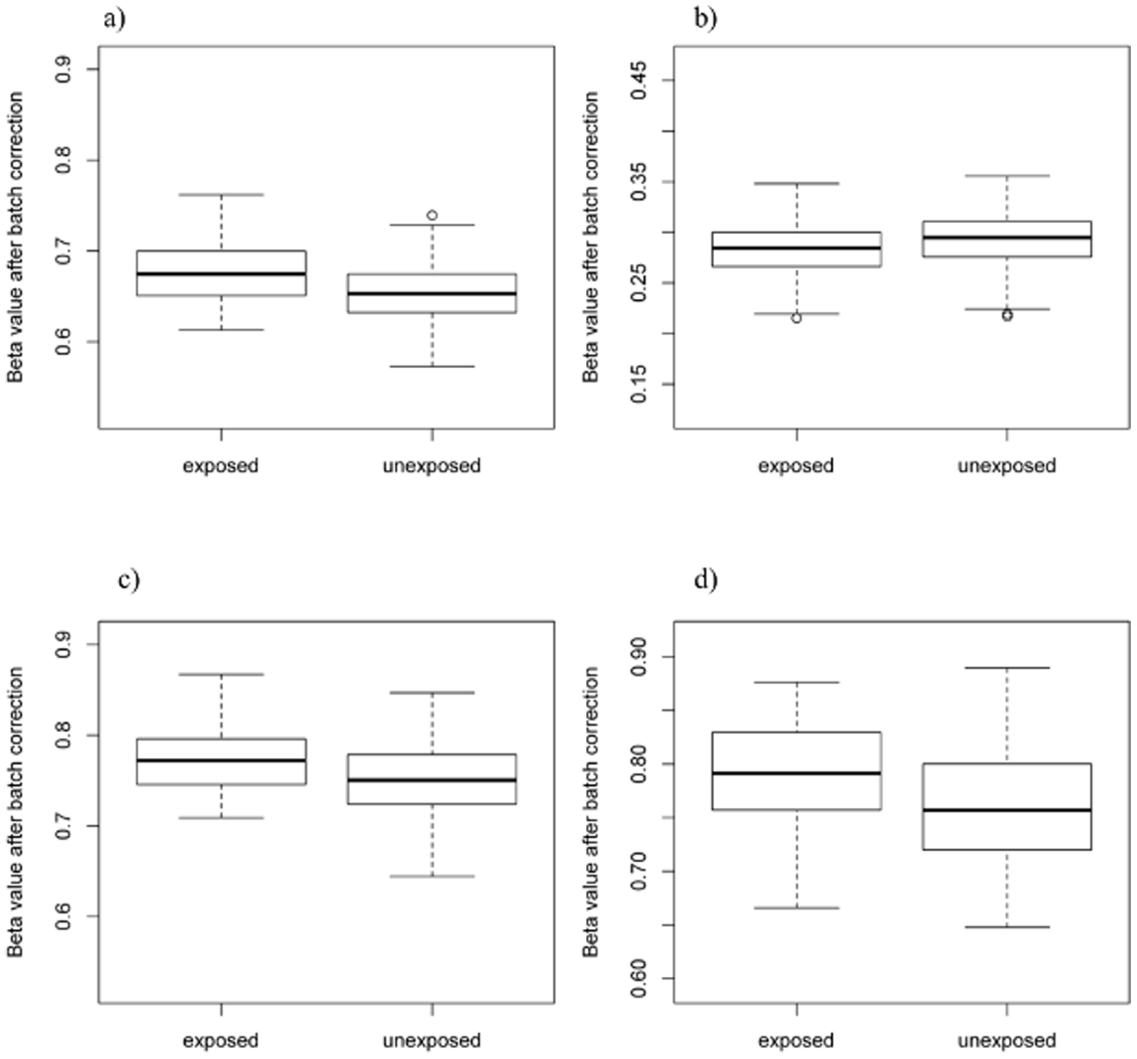
Boxplots showing the distribution of % CpG methylation after normalization and batch correction using COMBAT by IUS exposure for A) *C11orf52* (cg05697249), B) *XPNPEP1* (cg09352789), C) *FRMD4A* (cg25464840), and D) *PPEF2* (cg14724265) in 526 Asthma BRIDGE samples.

We evaluated the 19 CpGs in two replication datasets (Tables S1, S2 in File S1). In the Asthma BRIDGE replication population, three of the 19 loci were not available. Of the remaining 16, four loci had nominal p-values of less than 0.05: *XPNPEP1* (p = 0.01), *PPEF2* (p = 0.003), *FRMD4A* (p = 0.01), *C11orf52* (p = 0.04) (Table 3, Figure 1). *XPNPEP1* and *PPEF2* remained statistically significant after FDR adjustment for multiple testing. Moreover, the results were consistent if we further restricted this population to asthmatics only, to be more directly comparable to the CAMP population, suggesting that the maternal smoking effects on these genes are not restricted to asthmatic children. In MoBa, one locus was not found in that dataset and four loci had nominal p-values of less than 0.05: *FRMD4A* (p = 0.0009), *C11orf52* (p = 0.001), *SMPD3* (p = 0.02), and *CRYGN* (p = 0.05) (Table 3). *FRMD4A* and *C11orf52* remained significant after further adjustment for multiple testing. Interestingly, for some of these genes multiple CpG loci within the gene exhibited similar effect sizes and were located near the transcription start sites for *FRMD4A, CRYGN,* and *PPEF2* (Tables S3, S4 in File S1).

**Table 3:** Replication results from beta regression and linear regression models in Asthma BRIDGE and MoBa for the 19 CpG loci identified in CAMP.

			ABRIDGE (N = 526)		ABRIDGE Asthma only (N = 332)		Difference in methylation**		
Probe ID	Symbol	Chr	β*	FDR p-value	β*	FDR p-value	CAMP	ABRIDGE	MoBa
cg09352789	XPNPEP1	10	−0.07	0.04	−0.08	0.12	−0.01	−0.01***	−0.004
cg14724265	PPEF2	4	0.11	0.04	0.12	0.12	0.03	0.02***	−0.009
cg25464840	FRMD4A	10	0.08	0.07	0.08	0.18	0.02	0.02	0.03***
cg05697249	C11orf52	11	0.06	0.14	0.06	0.22	0.02	0.01	.02***
cg16184943	ZNF280B	22	−0.05	0.20	−0.06	0.22	−0.02	−0.01	0
cg04112019	IGF2AS	11	−0.05	0.29	−0.07	0.22	0.01	−0.003	−0.01
cg13473383	ZDHHC5	11	−0.04	0.29	−0.06	0.22	−0.01	−0.01	−0.001
cg20773127	ENPEP	4	0.04	0.29	0.03	0.63	0.02	0.01	0.003
cg14580737	RFXANK	19	−0.05	0.37	−0.06	0.37	−0.02	−0.004	0.004
cg22830895	CRYGN	7	−0.02	0.84	−0.02	0.69	0.03	−0.01	0.02
cg01058368	CDH10	5	0.0004	0.99	−0.01	0.90	0.02	0	0.01
cg07499072	FST	5	−0.002	0.99	−0.01	0.90	0.00	0	−0.003
cg09143663	BACH1	21	0.01	0.99	0.05	0.48	0.02	0.001	0.01
cg10556064	SMPD3	16	−0.01	0.99	−0.002	0.96	0.02	−0.001	0.02
cg20588045	PCDH15	10	−0.01	0.99	−0.01	0.90	0.02	−0.002	−0.002
cg24956866	CALD1	7	0.001	0.99	0.03	0.63	0.02	0.002	0.01
cg00169548	BAZ1A	14	n/a	n/a	n/a	n/a	−0.02	n/a	n/a
cg10493739	TMEM38B	9	n/a	n/a	n/a	n/a	0.00	n/a	−0.001
cg20555507	TRPM3	9	n/a	n/a	n/a	n/a	0.01	n/a	0.01

* coefficient from beta regression adjusted for age, sex, and clinic and cell type.

** coefficient from linear regression model illustrating difference in methylation level comparing IUS exposed to unexposed.

*** FDR corrected p- value <0.05.

In the initial screening analysis, the model was adjusted only for sex, age clinic, and cell type. We conducted additional analyses to evaluate whether the association was confounded by other potentially relevant factors. No evidence for confounding was noted for income, education, parental history of asthma, asthma severity, or exposure to paternal smoking in childhood (data not shown). As expected, report of exposure to maternal smoking during childhood was strongly associated with *in utero* smoke exposure, reported in 57 (88%) of 65 *in utero* exposed children. Evidence for collinear association by maternal smoking in childhood was present for some but not all of the loci, in some cases strengthening the association and in some cases decreasing the association for prenatal tobacco smoke exposure (Table S5 in File S1). For example, adjustment for childhood smoke exposure had little to no effect on the estimate for prenatal smoke exposure for *C11orf52* and *PPEF2.* However, adjustment for childhood smoke exposure attenuated the effect estimates for *FRMD4A* and *XPNPEP1.*

The direction and magnitude of effect estimates for IUS exposure was consistent in all datasets for all loci. Interestingly, *FRMD4A* and *C11orf52* showed consistently strong effect estimates across all three diverse populations, regardless of whether the children were asthmatic, and regardless of the timing of sample collection. In fact, the use of cord blood in the MoBa replication dataset lends support to the conclusion that the effects on DNA methylation in *FRMD4A* and *C11orf52* are due to prenatal rather than childhood smoke exposure.

Lastly, in the Asthma BRIDGE replication population, we evaluated the relation between DNA methylation and expression for *XPNPEP1*, *PPEF2*, and *FRMD4* as well as the association between IUS and expression. *C11orf52* could not be evaluated as there was no associated mRNA transcript. Though expression profiles in whole blood were available for 526 subjects, we did not observe significant associations between methylation and expression for the three tested genes. However, expression of two of these genes (*PPEF2* and *XPNPEP1*) was significantly decreased in subjects with IUS exposure compared to those without IUS exposure (β = −0.02, p = 0.02 for *PPEF2*; and β = −0.05, p = 0.01 for *XPNPEP1*), demonstrating that exposure was associated both with changes in both methylation and expression in the same sample.

## Discussion

In a comprehensive investigation of IUS exposure and DNA methylation in the offspring, we identified 19 CpG loci in whole blood significantly associated with IUS in asthmatic children, two of which (*FRMD4A* and *C11orf52*) were replicated in two additional independent populations and four others that warrant potential further investigation.

*FRMD4A* encodes a scaffolding protein involved in activation of Arf6, which in turn, plays a role in membrane trafficking, junctional remodeling and epithelial polarization. [22] Our observation of increased DNA methylation in *FRMD4A* in whole blood samples from children exposed to maternal smoking is consistent with an experimental study showing hypermethylated *FRMD4A* in MCF-7 cells treated with benzo(a)pyrene (BaP), a common polycyclic aromatic hydrocarbon that is a constituent of cigarette smoke. [23] Moreover, *FRMD4A* was recently identified from a GWAS study in Asian populations and validated in European and American populations as a gene associated with nicotine dependence. [24] IUS exposure is also associated with nicotine dependence in offspring. [25], [26] These observations raise the intriguing possibility that cigarette-smoke-induced epigenetic modification of the *FRMD4A* gene plays a role in conferring increased risk of nicotine dependence in offspring of mothers who smoke during pregnancy. Beyond nicotine dependence, altered DNA methylation of *FRMD4A* may have the potential to affect other downstream health outcomes, since the gene has recently been implicated as a risk factor for both Alzheimer’s disease and squamous cell carcinoma. [27], [28]

Very little is currently known about the other replicated locus within *C11orf52*. *C11orf52* describes an uncharacterized protein that is expressed in the lung and which has been associated with increased phosphorylation in non-small cell lung cancer tumor compared to normal tissue samples. [29] However, the *C11orf52* locus also overlaps with heat shock 27kDa protein 2 (*HSPB2*). *HSPB2* is a stress-inducible small heat shock protein that plays a role in airway smooth muscle (ASM) cell remodeling. *HSPB2* undergoes rapid phosphorylation in the ASM cell thereby stabilizing the cytoskeletal scaffolding and decreasing the rate of microstructural reorganization. [30] ASM remodeling plays a role in airway hyperresponsiveness and is a cardinal feature of asthma. [31] Moreover, particulate matter exposure was shown to stimulate reactive oxygen species generation in human lung vascular endothelium, resulting in increased *HSPB2* phosphorylation and a marked disruption in endothelial cell barrier function via cytoskeletal rearrangement, key elements for lung homeostasis. [32] Our observation of an IUS-induced change in CpG methylation level in a locus within *HSPB2* in asthmatic children raises the interesting question of whether these changes directly affect the function of or phosphorylation of *HSPB2* and could thus play a role in airway remodeling.

*XPNPEP1* and *PPEF2* are two genes that may also be of interest as they were significant in both the CAMP and Asthma BRIDGE populations. However, little is known about these genes. *XPNPEP1* located on chromosome 10, encodes the human soluble aminopeptidase P (APP) which is an aminoacylprolyl hydrolase. [33] APP is a key enzyme in the renin-angiotensin and kininogen-kinin hormonal systems and has been associated with oral contraceptive use. [34] Specifically APP degrades bradykinin, a blood pressure regulator peptide, and has been linked to myocardial infarction. [35] *PPEF2* is a protein phosphatase with EF-hand domain whose function is not well understood. *PPEF2* has also been implicated in dendritic cell development in a murine model [36] and with schizophrenia in a small population. [37] *PPEF2* expression correlates with stress protective responses, cell survival, growth and proliferation and is a negative regulator of apoptosis signal regulating kinase-1 (ASK1), important in cancer, cardiovascular, and neurodegenerative diseases. [38], [39] Our observation that IUS was associated with decreased expression in *PPEF2* may not be surprising, given *PPEF2*’s purported role in stress response. However, we provide some of the first evidence for an association between IUS exposure and expression and CpG methylation in both *PPEF2* and *XPNPEP;* thus, a much greater understanding of the biological roles of these genes is necessary before we can understand the health implications of IUS on them.

*SMPD3* and *CRYGN* are additional genes for which DNA methylation was significantly associated with IUS exposure in both the CAMP and MoBA, but did not meet the stringent criteria for replication after adjustment for multiple testing. *SMPD3* is the primary regulator of ceramide biosynthesis, and plays a pivotal role in the control of late embryonic and postnatal development such that defects in the gene lead to dwarfism and pituitary hormone deficiency. [40], [41] *SMPD3* has also been implicated in macrophage differentiation and leukemia. [42] Given *SMPD3*’s crucial role in development and its expression in neurons in the central nervous system, our observed association with IUS raises intriguing questions about the biological mechanisms underlying smoking’s affect on prenatal growth. Lastly, *CRYGN* encodes a crystallin, one of the main structural proteins in the eye. [43] Very little research on *CRYGN* currently exists, thus the implications of our association with IUS with regard to developmental biology are largely unknown.

In this study, the associations between IUS and DNA methylation of several genes were observed using questionnaire-based recall of maternal smoking as well as measured cotinine levels in cord blood. While questionnaire-based recall of maternal smoking history is susceptible to recall bias, the positive findings reported in a previous analysis in the CAMP cohort supports the validity of the retrospective history. [44] DNA methylation was measured both at birth in cord blood (MoBa) and in whole blood at age 9 (CAMP) and in adults (Asthma BRIDGE). Our robust results suggest the IUS effects on DNA methylation may persist over years at least for some genes. An additional strength of our use of cord blood for replication is that we remove potential confounding effects of childhood maternal or paternal smoking exposure, and can more confidently conclude the observed effects are due to *in utero* exposure. On the other hand, because our primary and replication studies were performed using whole blood collected at different time points, we may have missed transient yet important effects on DNA methylation.

DNA methylation was measured in whole blood, composed of a variable mixture of circulating cell types. In theory, a shift in cell populations caused by IUS, rather than differences in DNA methylation, could explain our observed results. In order for this to be true, however, IUS would have to alter the proportions of cell populations not only at birth but 9 or more years later. To shed further light on this possibility, we estimated 6 cell types using the method of Houseman et al [19] and included these estimated cell types as covariates in the regression model. Additionally, we tested whether IUS exposure was associated with cell type and found no association. Given these results, it is unlikely that differences in cell types are accounting for our observed associations. Because we evaluated DNA methylation in whole blood, we do not know whether these same observations would be apparent in other tissue or cell types of interest.

DNA methylation in our loci was not highly correlated with expression in whole blood. However, IUS exposure was significantly associated with decreased expression levels in *PPEF2* and *XPNPEP1.* One reason for the lack of direct correlation between methylation and expression may be because the methylated loci for *PPEF2* and *XPNPEP1* lie within gene bodies, and not the promoters typically associated with expression levels, [45] and thus may be only one piece in a complex regulatory network. An additional reason may be that these genes are functionally relevant only *in utero* or shortly after birth, during the time at which maternal smoking exposure occurred. While the methylation marks have endured and represent a biomarker of past exposure, the functionality of the gene may change with developmental time period and tissue.

Lastly, we acknowledge that the use of the HM27 assay is not an epigenome-wide interrogation of DNA methylation. Nevertheless, the HM27 assay served our hypothesis well, since we hypothesized IUS exposure would be associated with CpG methylation in promoter regions of genes that regulate expression.

In summary, our results showing an increase in DNA methylation level at two loci in *FRMD4A* and *Cllorf52* add to the growing evidence that IUS exposure in humans has epigenetic consequences for the offspring, some of which may persist throughout childhood. Though the long term health implications of these loci are currently unknown and require further investigation, their implication in nicotine dependence and lung homeostasis suggests their potential contribution to the development of disease in later life.

## Supporting Information

File S1 **The Asthma BRIDGE Consortium Authorship list.**
(DOCX)
