# Correction: Prenatal Tobacco Smoke Exposure Is Associated with Childhood DNA CpG Methylation

**DOI:** 10.1371/journal.pone.0112422

**Published:** 2014-10-28

**Authors:** The PLOS ONE Staff

## Notice of Republication

This article was republished on October 14, 2014, to correct errors that were introduced during the typesetting process. Please download this article again to view the correct version. The originally published, uncorrected article and the republished, corrected article are provided here for reference.

## Supporting Information

File S1 Originally published, uncorrected article.
(PDF)

File S2 Republished corrected article.
(PDF)
