# Supplementary material for: Prenatal Tobacco Smoke Exposure Is Associated with Childhood DNA CpG Methylation
Source: PLoS One. 2014 Jun 25;9(6):e99716. doi: 10.1371/journal.pone.0099716 (PMC4070909; doi:10.1371/journal.pone.0099716)
Supplement: File S1 — The Asthma BRIDGE Consortium Authorship list. (DOCX) [file pone.0099716.s001.docx]

**Supplement**

**Appendix**

**The Asthma BRIDGE Consortium Authorship list**

**Childhood Asthma Management Program (CAMP) Genetics Ancillary Study**

Brigham and Women’s Hospital, Harvard Medical School, Boston MA

Benjamin A. Raby, MD, MPH (PI), Scott T. Weiss, MD, MSc (PI); Vincent Carey, PhD; Weiliang Qiu, PhD; Roxanne Kelly, BSc; Jody Sylvia Senter, MS; John Ziniti, BSc; Diana Tubbs; Brooke Schumann; Damien Croteau-Chonka, PhD.

**Childhood Asthma Research and Education (CARE) Network**

Arizona Respiratory Center, University of Arizona (Coordinating Center)

Fernando Martinez, MD, (PI); Wayne Morgan, MD; James Goodwin, PhD; Anthony Bosco, PhD (also at Telethon Institute for Child Health Research, Centre for Child Health Research, University of Western Australia); Monica Vasquez, MPH; Rosemary Weese, RN; Silvia Lopez, RN; Jesus Wences, BS; Monica Varela, LPN; Janette Priefert; Katherine Chee; Samira Ehteshami, BS; Xiaobing Liu, BS.

**National Jewish Health, Denver CO**

Andy Liu, MD (PI); Allison Schlitz, BA; Julie Henley, CCRC; D. A. Sundström, BA; Melanie Phillips, BS; Sakari Graves,  BA; Phillip Lopez,   BS; Liliana Soto, CRC.

**University of Wisconsin–Madison, Maddison WI**

Robert F. Lemanske, Jr., MD (PI); Theresa W. Guilbert, MD; Sarah Sund, BS, MT (ASCP); Tiffany Huard, BS, CCRC; Elizabeth A. Schwantes, BS;

**Washington University School of Medicine, St. Louis MO**

Robert C. Strunk, MD (PI); Tina Norris (was Oliver) CCRP, CRT; Wanda Caldwell, RRT; Cynthia Moseid;

**Chicago Asthma Genetics (CAG) Study**

University of Chicago, Chicago IL

Carole Ober, PhD (PI); Dan Nicolae, PhD; Julian Solway MD; Krishnan Jerry, MD, PhD; White Steve, MD; Kyle Hogarth, MD; John McConville, MD; Rebecca Anderson, MS; Myers Rachel, PhD.

**Genomic Research on Asthma in the African Diaspora (GRAAD)**

Johns Hopkins University, Baltimore MD

Kathleen C. Barnes, PhD (PI); Nadia Hansel, MD, MPH; John T. Schroeder, PhD; Chris Cheadle, PhD; Dmitry Grigoryev, MD, PhD; Rasika A. Mathias, ScD; Alan E. Berger, PhD; Jinshui Fan, MD, PhD; Candelaria Vergara, MD, MSc; Anja Bieneman; Cassandra Foster; Tonya Watkins, MS; Susan Balcer Whaley, MPH; Li Gao, MD, PhD; Joseph Potee, MS.

**Mexico City Childhood Asthma Study (MCCAS)**

National Institute of Environmental Health Sciences, National Institute of Public Health of Mexico, Hospital Infantil de Mexico Federico Gomez

Stephanie J London, MD, DrPH (PI); Albino Barraza Villarreal , MSc, DrPH; Leticia Hernandez Cadena, MSc, DrPH; Efrain Navarro Olivos, MD, MSc; Isabelle Romieu, MD, MPH, DrS; Juan Jose Sienra Monge, MD; Blanca Estela del Río Navarro, MD; Isabelle García; Cynthia Hernandez.

**Children’s Health Study (CHS)**

University of Southern California, Los Angeles CA

Frank D. Gilliland, MD, PhD (PI); Jim Gauderman, Ph.D., Talat Islam, MBBS, PhD; Carrie V. Breton, ScD; Muhammad T. Salam, MBBS, PhD; Kimberly D. Siegmund, PhD; Xinhui Wang, MS

**The Norwegian Mother and Child Cohort Study (MoBa) Epigenetics Project**

National Institute of Environmental Health Sciences, Norwegian Institute of Public Health, University of Bergen.

Stephanie J. London, MD, DrPH; Bonnie R. Joubert, PhD; Shyamal Peddada, PhD; Wenche Nystad PhD, Siri Håberg, MD, PhD; Stein Emil Vollset, PhD, Per Magne Ueland, PhD; Øivind Midtun, PhD.

**Methods**

*Study Population*

**1) The Childhood Asthma Management Program (CAMP)** was a multi-center, randomized, double-masked, clinical trial designed to determine the long-term effects of three inhaled treatments for mild to moderate asthma in children: 1) budesonide, (an inhaled corticosteroid) used daily with as-needed albuterol (a short-acting beta agonist bronchodilator); 2) nedocromil, a non-steroidal anti-inflammatory agent used daily with as-needed albuterol; and 3) placebo with as-needed albuterol. CAMP enrolled 1,041 children ages 5 to 12 yr with mild to moderate chronic asthma over a 23-month period. Entry criteria and asthma definition included (1) a doctor diagnosis of asthma; (2) confirmed evidence of airway hyperresponsiveness as defined as provocative concentration of methacholine causing a 20% reduction in FEV1 (PC20) < 12.5 mg/ml; and (3) asthma symptoms and/or medication use for 6 mo or more in the previous year, and that children have chronic asthma as evidenced by one or more of the following findings for at least 6 mo in the year prior to enrollment: (i) asthma symptoms at least twice a week, (ii) use of an inhaled bronchodilator at least twice a week, and (iii) daily asthma medication. Study participants with evidence of severe or unstable asthma, or with other clinically significant conditions were excluded from enrollment. 968 (93%) of CAMP children and 1518 (77%) of their parents participated in a Genetics Ancillary Study. The CAMP Continuation Study/ Phase 2 (CAMPCS/2) was a 4-year observational follow-up study of children enrolled in the CAMP Study. In March 2006, subjects were invited to participate in a new protocol to collect biologic materials for genomic investigation. Consent was obtained for blood draw, generation of cell lines, isolation of DNA and RNA, and use of these materials for asthma related research. At these CAMP CS/2 visits, interim history questionnaires were administered, and spirometry was measured. Through these efforts, 618 cell lines and 700 whole blood RNA samples were collected. We contacted the subset of subjects who provided these samples to obtain additional informed consent for inclusion of these samples in Asthma BRIDGE, including explicit permission to make the cell lines and accompanying datasets available to researchers through BioLINCC. 572 individuals provided such consent for whom both cell lines and whole blood RNA samples were available.

**2) Childhood Asthma Research and Education (CARE) Network:** The Childhood Asthma Research and Education (CARE) Network was established in 1999 by the National Heart, Lung and Blood Institute to accelerate clinical trials in pediatric asthma. The various CARE clinical trials enrolled children between the ages of 1-18 years at the time of their study participation and who had a confirmed diagnosis of asthma or wheezing illness. Male and female non-smoking children were enrolled into at least one of eight protocols in the Childhood Asthma Research and Education Network (CARE). Approximately 900 childhood subjects of well-distributed age, race, ethnicity, and gender participated in an exceptionally well-phenotyped group of subjects with a range of asthma that includes toddlers with wheezing illnesses, as well as older children 6-18 years old with mild to moderate intermittent and persistent asthma. Consent for blood draw for genetic studies was obtained from most CARE participants. DNA and whole-genome genotype data is available for 436 parent-child trios ascertained through participants in CARE trials. These subjects have been phenotyped using standardized protocols, with spirometric evaluation by ATS standards, methacholine bronchoprovocation, exhaled NO measurements, and data on asthma exacerbation.

The CARE protocols include:

The Prevention of Early Asthma in Kids (**PEAK**) study was a 3-year clinical trial to determine whether the future development of asthma could be altered by starting an inhaled corticosteroid early in the life of Asthma Predictive Index (API) positive, preschool children (2-4 years of age). 126 eligible randomized subjects participated in PEAK.

The Characterizing the Response to a Leukotriene Receptor Antagonist (LTRA) and an Inhaled Corticosteroid (**CLIC**) study was a 20-week clinical trial in children 6-18 years of age to examine the genetic and other physiological characteristics of the subject’s response to montelukast and an inhaled steroid (ICS). 75 eligible randomized subjects participated in CLIC.

The Pediatric Asthma Controller Trial (**PACT**) was designed to determine what is the first line choice in controller therapy: inhaled corticosteroid (ICS) alone; ICS in combination with inhaled Long-Acting Beta Agonist (LABA); or a LTRA alone for 6-14 year-old children with mild-moderate persistent asthma. Study duration included 48 months of treatment. 122 eligible randomized subjects participated in PACT.

The Acute Intervention Management Strategies (**AIMS**) was a study comparing the effectiveness of three treatments at the onset of respiratory symptoms: high-dose ICS plus albuterol; LTRA once daily plus albuterol; or albuterol alone in increasing episode-free days among 12-59 month old children with recurrent severe wheezing. 122 eligible randomized subjects participated in AIMS.

The Montelukast or Azithromycin for Reduction of Inhaled Corticosteroids in Childhood Asthma (**MARS**) study was designed to determine whether or not the use of a LTRA (montelukast) or macrolide (azithromycin) will provide a steroid sparing effect in 6-17 year-old children with asthma who require moderate to high dose ICS despite receiving concomitant LABA. 18 eligible randomized subjects participated in MARS.

All non-Hispanic white CARE participants with GWAS genotype data were invited to participate in Asthma BRIDGE, from four clinical CARE centers: the University of Arizona, Tucson, the University of Wisconsin, and the National Jewish Medical and Research Center, and Washington University, St. Louis.

**3) Chicago Asthma Genetics (CAG) Study:** The Chicago Asthma Genetics Study (C.A.G.) was designed to identify genes that influence risk for asthma or asthma-related phenotypes in families representing diverse ethnic groups. The University of Chicago studied European American and African American a) families ascertained through affected sib pairs, b) affected children and their parents, c) adults and children with severe persistent asthma, and d) non-asthmatic control subjects (over the age of 18 years). Samples a-c were recruited in the adult and/or pediatric asthma clinics at University of Chicago Hospital; controls have been recruited from the medical center at large. In both of these studies, asthma was diagnosed as follows: 1) presence of at least 2 of 3 symptoms (cough, wheeze, shortness of breath), 2) doctor’s diagnosis of asthma, and 3) either 20% fall in baseline FEV_1_ after inhalation of 25 mg/ml methacholine or 15% improvement of baseline FEV_1_ after inhalation of albuterol. All subjects were at least 6 years of age. Severe persistent asthma is defined as 1) FEV_1_ < 60% predicted, 2) using either oral steroids withor without current symptoms or inhaled steroids with current symptoms, 3) nocturnal symptoms, and 4) either 15% improvement of baseline FEV_1_ after inhalation of albuterol or 15% time-related reversibility on medications. Individuals with the following conflicting diagnoses were excluded from these study: birthweight <2 kb, congenital pulmonary disease, conflicting pulmonary diagnosis, TB, severe cardiac disease, isolated occupational induced asthma, systemic vasculitis including the lungs. All eligible asthmatics (and their healthy relatives in the family studies) underwent the following: spirometry to assess lung function, methacholine challenge studies if their baseline FEV_1_ was ³70% predicted, airway reversibility studies if their baseline FEV_1_ was <70% predicted, skin prick testing to 14 allergens, blood sampling for serum IgE studies, eosinophil counts, and DNA extractions. Control subjects were recruited at the University of Chicago Medical Center and were ≥18 years of age and had a negative personal and family history of asthma. The CAG cohort was genotyped using the Illumina Infinium™ II HumanHap1M BeadChip (Illumina Inc.), in collaboration with Drs. D. Meyers and E. Bleecker (Wake Forest University). For the Asthma BRIDGE initiative, we invited all participants with the GWAS genotyping, with the goal of recruiting 100 subjects.

**4) Children’s Health Study (CHS):** The CHS is an on-going prospective study that has involved over 11,000 school children living in southern California. The original study consisted of cohorts of 6000 4^th^, 7^th^, and 10^th^ grade children from 12 Southern California communities. A new cohort of 5341 kindergarten and 1^st^ grade children was enrolled in 2002 to examine relationships of asthma with air pollution as part of a P01 supported by NIEHS. These children were enrolled from 13 communities selected to represent the full range and available combinations of regional pollutants in the area. All participants are surveyed annually during school visits and through questionnaires to assess personal and parental medical history, including doctor-diagnosed asthma. Children are characterized as having doctor-diagnosed asthma at study entry or during active follow-up (asthmatics), or as never having a diagnosis of asthma (non-asthmatics). Self-reported asthma status has been validated in our cohort by a comparison to medical records and spirometry, with more than 90% concordance. A rich dataset of measured and modeled hourly, daily, and annual outdoor air pollution data throughout their childhood is also available. For the Asthma BioiRepository initiative, 250 former CHS non-Hispanic white and Hispanic white participants greater than 18 years of age (125 with asthma and 125 controls) were re-contacted and visited for the purpose of collected a blood sample and administering a health questionnaire. This study oversampled asthmatics from the potential pool of over 6,000 CHS subjects to provide the proposed asthma case-control sample.

**5) Genomic Research on Asthma in the African Diaspora (GRAAD):** This study is comprised of adult and pediatric African American asthma cases and non-asthmatic controls (N=998) recruited through Johns Hopkins University and/or Howard University, in the Baltimore-Washington, D.C. metropolitan area (mean age 29.55 ± 18.10). The estimated proportion of African ancestry is similar for African American cases and controls (72.3% and 72.5%, respectively). GRAAD asthmatics are younger (mean 23.9 ± 17.8 years) compared to controls (35.2 ± 16.5 years), as there was a deliberate decision to favor adults in the control group to minimize including controls with some potential for developing asthma. Among all cases, asthma was defined as both a reported history of asthma and a documented history of physician-diagnosed asthma (past or current). A standardized questionnaire based on either the ATS RHQ or International Study of Asthma and Allergy in Childhood was administered by a clinical coordinator. Controls were administered a standardized questionnaire and determined to be negative for a history of asthma, with the exception of 50 ascertained through a study of human pigmentation, for which asthma status was explicitly determined, although “known clinical disease” was among the exclusion criteria. Genome-wide SNP genotyping data was generated using the Illumina Infinium™ II HumanHap650Y BeadChip v.1.0 (Illumina Inc.) 848 GRAAD participants (85% of the case-control group) were eligible for, and invited to participate in, the proposed sample collections for the Asthma BRIDGE initiative, with the goal of sampling 200 subjects.

**6) Mexico City Childhood Asthma Study:** The Mexico City Childhood Asthma Study is a case-parent triad study of asthmatic children and their parents enrolled at the allergic referral clinic at a large public pediatric hospital in Mexico City. 600 case-parent triads were enrolled. Children had skin testing to a battery of 24 aeroallergens and over 90% of children tested positive. Cases were clinically diagnosed by a pediatric allergist based on symptoms and response to treatment. Most children underwent pulmonary function testing. A total of 492 trios had genome wide genotyping with the Illumina 550K V3 chip. A subset of 200 children with available GWAS data were recruited for the Asthma BRIDGE initiative. Sample collection was conducted by home visits. Children are underwent spirometry before and after bronchodilator and exhaled nitric oxide testing. Human Subjects approval for the MCCAS study was obtained from both the National Institute of Public Health in Mexico, and from the NIH Ethics Office (Protocol number OH99-E-N028).

*DNA methylation*

Laboratory personnel performing DNA methylation analysis were blinded to study subject information. DNA was extracted from whole blood cells using the QiaAmp DNA blood kit (Qiagen Inc, Valencia, CA) and stored at -80 degrees Celcius. Samples were randomized onto 96-well plates. Five control samples were spotted on each plate, including a pooled sample of human white blood cell DNA, and a four-sample dilution series (10%, 35%, 60%, and 85% methylation) of mixtures of generally unmethylated sperm DNA and M.*Sss*I CpG methylase-treated sperm DNA. To evaluate the controls, all probes that had methylation values >0.65 in any of the seven 10% dilution samples were excluded, as these were considered to be constitutively methylated loci in sperm. The control samples were used to evaluate assay reproducibility and accuracy in the remaining 21,391 probes. There were a total of 7 replicates for each type of control. Spearman correlation coefficients were calculated for each set of replicates. B

ias (defined as bias of the probes to estimate % methylation from sample mixtures) was calculated as bias = mean - expected value.

Two micrograms of genomic DNA from each sample were treated with bisulfite using the EZ-96 DNA Methylation Kit™ (Zymo Research, Irvine, CA, USA), according to the manufacturer’s recommended protocol and eluted in 18 ul. A 3 ul aliquot of bisulfite converted DNA was removed to evaluate the quantity of the bisulfite-converted DNA, as well as the completeness of the bisulfite conversion, using a panel of quality control reactions as previously described. ^1^ All samples passed these quality control tests, and 5 µl of each sample was used in the Illumina HumanMethylation27 (HM27) assay as specified by the manufacturer.

The results of the HM27 assay were compiled for each locus as previously described ^2^ and were reported as beta (β) values, in which  β  is the ratio of the mean methylated signal intensity (M) over the sum of the mean methylated and unmethylated signal intensities (M+U) for each locus. Beta values range from 0 to 1, reflecting the fractional DNA methylation level of the CpG site. ^3^ Detection p-values for each data point were also calculated as described ^2^ and were computed using a panel of negative control signals to determine if the analytical target signal is indistinguishable from the signal intensities of the negative control probes. Data points with detection p-values > 0.05 were set to missing. The methylumi package version 2.4.0 using the R programming language, version R2.15.3.was used for analysis.

**References**

1. Campan M, Weisenberger DJ, Trinh B, Laird PW. Methylight. Methods Mol Biol. 2009; 507:325-337.

2. Noushmehr H, Weisenberger DJ, Diefes K, Phillips HS, Pujara K, Berman BP, et al. Identification of a cpg island methylator phenotype that defines a distinct subgroup of glioma. Cancer Cell. 2010; 17:510-522.

3. Bibikova M, Lin Z, Zhou L, Chudin E, Garcia EW, Wu B, et al. High-throughput DNA methylation profiling using universal bead arrays. Genome Res. 2006; 16:383-393.

**Figure Legend**

**Supplemental Figure S1.** The density distributions of 22,131 loci from the Illumina HM27k assay using a) raw beta values, b) Norm exponential (NE) background corrected values, and c) NE plus Quantile regression normalized values.

**Figure S1.**


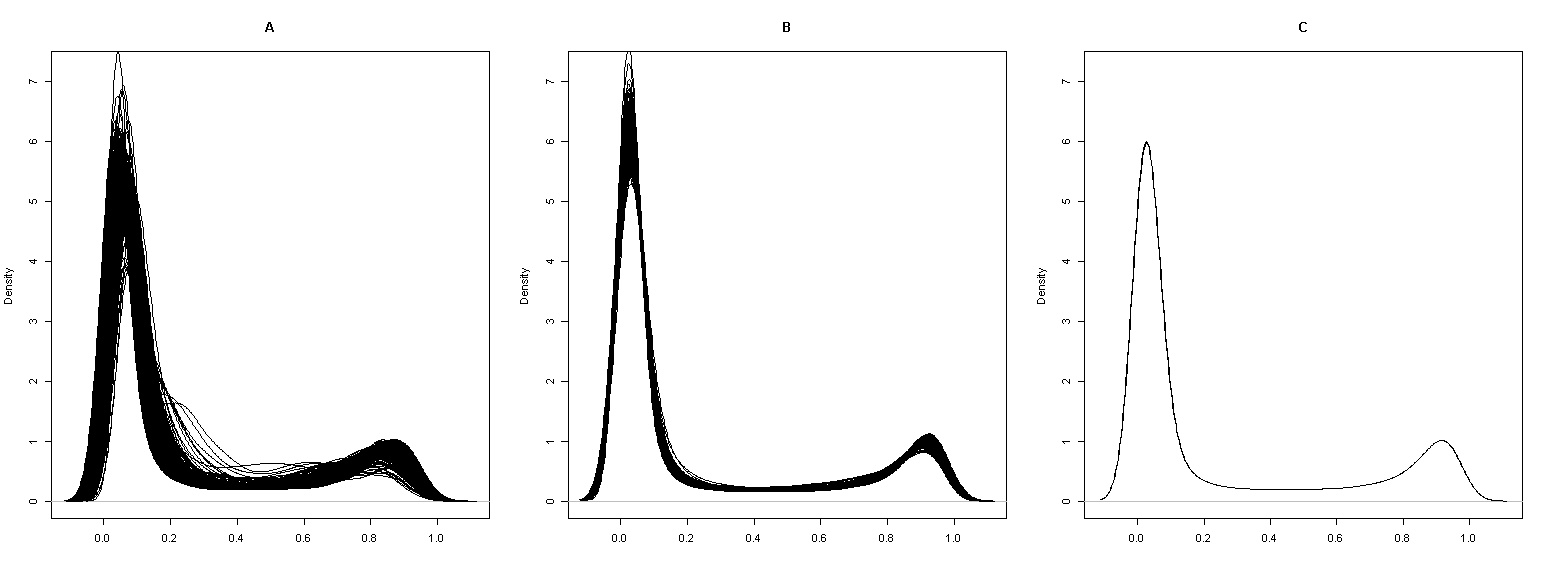


| **Table S1. Descriptive characteristics of the ABRIDGE study population (n=526)** | | | | | | | | |
| --- | --- | --- | --- | --- | --- | --- | --- | --- |
|  |  |  |  | ***Exposure to maternal smoking in utero*** | | | | |
|  |  | **Overall** |  | **No (N=462)** | | **Yes (N=65)** | |  |
| **Characteristics** | **level** | **Count** | **%** | **Count** | **%** | **Count** | **%** | **p-values*** |
| *In utero* tobacco smoke exposure |  | 51 | 9.7 |  |  |  |  |  |
| Male |  | 223 | 42.4 | 200 | 42.1 | 23 | 45.1 | 0.79 |
| Maternal history of asthma |  | 69 | 13.3 | 64 | 13.6 | 5 | 10.2 | 0.65 |
| Paternal history of asthma |  | 57 | 11.5 | 49 | 10.9 | 8 | 17.4 | 0.28 |
| Education | ≤ high school | 182 | 35.8 | 160 | 34.7 | 22 | 45.8 | 0.27 |
|  | some college | 158 | 31.0 | 144 | 31.2 | 14 | 29.2 |  |
|  | college degree | 236 | 33.2 | 157 | 34.1 | 12 | 25.0 |  |
| Annual income | < $30,000 | 202 | 42.9 | 176 | 41.8 | 26 | 53.1 | 0.36 |
|  | $30,001-$50,000 | 127 | 27.1 | 114 | 27.1 | 13 | 26.5 |  |
|  | $50,001-$75,000 | 73 | 15.5 | 67 | 15.9 | 6 | 12.2 |  |
|  | More than $75,000 | 68 | 14.5 | 64 | 15.2 | 4 | 8.2 |  |
| Clinic site | GRAAD,JH | 101 | 19.2 | 76 | 16.0 | 25 | 49.0 | <0.0001 |
|  | CAG/UAC | 41 | 7.8 | 36 | 7.8 | 5 | 9.8 |  |
|  | CHS/USC | 225 | 42.7 | 215 | 45.3 | 10 | 18.6 |  |
|  | CARE | 39 | 7.4 | 34 | 7.2 | 5 | 9.8 |  |
|  | MCCAS | 129 | 22.8 | 114 | 24.0 | 6 | 11.8 |  |
| Age (mean(SD)) |  | 27.1 | 10.9 | 26.3 | 10.0 | 34.5 | 15.6 | 0.0005 |
| *p-values are calculated using chi-sq test | | | | | | | | |

| **Table S2. Descriptive characteristics of the MoBa study population (n=1062)** | | | | | | | | |  |
| --- | --- | --- | --- | --- | --- | --- | --- | --- | --- |
|  |  |  |  | ***Exposure to maternal smoking in utero*** | | | | | |
|  |  | **Overall** |  | **No (N=926)** | | **Yes (N=136)** | |  | |
| **Characteristics** | **level** | **Count** | **%** | **Count** | **%** | **Count** | **%** | **p-values*** | |
| *In utero* tobacco smoke exposure |  | 136 | 12.8 |  |  |  |  |  | |
| Male |  | 566 | 53.3 | 492 | 53.1 | 74 | 54.1 | 0.851 | |
| Maternal history of asthma |  | 116 | 10.9 | 97 | 10.5 | 19 | 14.0 | 0.283 | |
| Paternal history of asthma |  | 64 | 6.0 | 58 | 6.3 | 6 | 4.4 | 0.513 | |
| Maternal education | ≤ high school | 78 | 7.3 | 328 | 35.4 | 93 | 68.4 | <0.0001 | |
|  | some college |  |  | 437 | 47.2 | 34 | 25.0 |  | |
|  | college degree | 170 | 16.0 | 161 | 17.4 | 9 | 6.6 |  | |
| *Maternal* age (mean(SD)) |  | 30.0 | 4.3 | 30.0 | 4.2 | 29.6 | 5.2 | <0.0001 | |
| *p-values are calculated using chi-sq test | | | | | | | | | |

| **Table S3. All CpG loci within the 19 genes significantly associated with IUS exposure in CAMP asthmatics (N=527)** | | | | | | |
| --- | --- | --- | --- | --- | --- | --- |
| **Probe ID** | **Symbol** | **Chr** | **Location** | **Distance to TSS** | **β*** | **Raw p-value** |
| cg14724265 | PPEF2 | 4 | 77042685 | 19 | 0.17 | <0.0001 |
| cg22476251 | PPEF2 | 4 | 77042725 | 19 | 0.01 | 0.63 |
| cg20773127 | ENPEP | 4 | 111616604 | 73 | 0.10 | 0.0001 |
| cg07290435 | CDH10 | 5 | 24680650 | 118 | 0.05 | 0.02 |
| cg01058368 | CDH10 | 5 | 24681244 | 401 | 0.12 | <0.0001 |
| cg01301664 | FST | 5 | 52811562 | 458 | 0.00 | 0.94 |
| cg07499072 | FST | 5 | 52812451 | 429 | 0.11 | <0.0001 |
| cg02382666 | CALD1 | 7 | 134113994 | 709 | -0.05 | 0.05 |
| cg24956866 | CALD1 | 7 | 134114669 | 34 | 0.10 | 0.0001 |
| cg14009688 | CALD1 | 7 | 134114782 | 77 | 0.07 | 0.02 |
| cg21035142 | CRYGN | 7 | 150767873 | 259 | -0.02 | 0.22 |
| cg22830895 | CRYGN | 7 | 150768409 | 376 | 0.11 | <0.0001 |
| cg20555507 | TRPM3 | 9 | 72926272 | 162 | 0.13 | <0.0001 |
| cg04725234 | TMEM38B | 9 | 107496460 | 166 | 0.02 | 0.26 |
| cg10493739 | TMEM38B | 9 | 107496802 | 256 | -0.07 | <0.0001 |
| cg05755354 | FRMD4A | 10 | 14412602 | 269 | 0.07 | 0.001 |
| cg25464840 | FRMD4A | 10 | 14412916 | 43 | 0.13 | <0.0001 |
| cg03808835 | PCDH15 | 10 | 56230951 | 105 | 0.04 | 0.17 |
| cg20588045 | PCDH15 | 10 | 56231102 | 44 | 0.10 | <0.0001 |
| cg09352789 | XPNPEP1 | 10 | 111672683 | 617 | -0.06 | <0.0001 |
| cg17093267 | XPNPEP1 | 10 | 111673644 | 342 | 0.03 | 0.52 |
| cg17773950 | IGF2AS | 11 | 2118174 | 49 | -0.01 | 0.77 |
| cg13791131 | IGF2AS | 11 | 2118468 | 133 | -0.03 | 0.30 |
| cg25574024 | IGF2AS | 11 | 2118470 | 135 | -0.03 | 0.60 |
| cg21237591 | IGF2AS | 11 | 2119086 | 168 | 0.00 | 0.96 |
| cg10501065 | IGF2AS | 11 | 2121552 | 2634 | 0.00 | 0.89 |
| cg04112019 | IGF2AS | 11 | 2121712 | 2794 | 0.07 | <0.0001 |
| cg20792294 | IGF2AS | 11 | 2122001 | 3083 | -0.02 | 0.39 |
| cg12322132 | IGF2AS | 11 | 2122232 | 3314 | -0.02 | 0.43 |
| cg16817891 | IGF2AS | 11 | 2122403 | 3485 | 0.02 | 0.48 |
| cg11005826 | IGF2AS | 11 | 2122537 | 3619 | 0.01 | 0.76 |
| cg13473383 | ZDHHC5 | 11 | 57190954 | 1095 | -0.08 | <0.0001 |
| cg18493899 | ZDHHC5 | 11 | 57192075 | 24 | -0.03 | 0.16 |
| cg08775230 | C11orf52 | 11 | 111294703 | 44 | -0.01 | 0.80 |
| cg05697249 | C11orf52 | 11 | 111294903 | 6232 | 0.09 | <0.0001 |
| cg07414384 | BAZ1A | 14 | 34414418 | 185 | 0.02 | 0.28 |
| cg00169548 | BAZ1A | 14 | 34414883 | 179 | -0.10 | 0.00003 |
| cg00891541 | SMPD3 | 16 | 67038988 | 921 | 0.09 | 0.0002 |
| cg10556064 | SMPD3 | 16 | 67038990 | 919 | 0.10 | <0.0001 |
| cg22116290 | SMPD3 | 16 | 67039469 | 440 | 0.05 | 0.01 |
| cg23758485 | SMPD3 | 16 | 67039703 | 206 | 0.05 | 0.07 |
| cg15201635 | SMPD3 | 16 | 67040138 | 227 | 0.03 | 0.15 |
| cg19297232 | SMPD3 | 16 | 67040522 | 611 | -0.03 | 0.46 |
| cg17217677 | SMPD3 | 16 | 67040695 | 784 | 0.00 | 0.96 |
| cg14580737 | RFXANK | 19 | 19162780 | 1227 | -0.10 | 0.0001 |
| cg09143663 | BACH1 | 21 | 29592287 | 803 | 0.08 | 0.00004 |
| cg26635603 | BACH1 | 21 | 29593762 | 153 | -0.01 | 0.63 |
| cg16184943 | ZNF280B | 22 | 21193293 | 211 | -0.09 | <0.0001 |
| * coefficient from beta regression adjusted for age, sex, and clinic and cell type | | | | | | |

| **Table S4. All CpG loci within the 19 genes significantly associated with IUS exposure in ABRIDGE subjects (N=526)** | | | | | | | |  |
| --- | --- | --- | --- | --- | --- | --- | --- | --- |
| **Probe ID** | **Symbol** | **Chr** | **Location** | **Distance to TSS** | **Mean methylation level** | **β*** | **Raw p-value** | |
| cg12289476 | PPEF2 | 4 | 76781503 | 36032 | 0.89 | 0.04 | 0.36 | |
| cg08783845 | PPEF2 | 4 | 76796984 | 20551 | 0.92 | -0.06 | 0.10 | |
| cg17445155 | PPEF2 | 4 | 76807543 | 9992 | 0.84 | -0.08 | 0.22 | |
| cg25987417 | PPEF2 | 4 | 76823448 | 232 | 0.88 | -0.07 | 0.15 | |
| cg14724265 | PPEF2 | 4 | 76823661 | 19 | 0.72 | 0.06 | 0.20 | |
| cg22476251 | PPEF2 | 4 | 76823701 | 19 | 0.92 | 0.03 | 0.33 | |
| cg02362409 | PPEF2 | 4 | 76823713 | 31 | 0.95 | -0.02 | 0.63 | |
| cg18327952 | PPEF2 | 4 | 76823763 | 81 | 0.85 | 0.04 | 0.16 | |
| cg21177165 | PPEF2 | 4 | 76824682 | 1000 | 0.90 | -0.04 | 0.50 | |
| cg08733482 | ENPEP | 4 | 111395729 | 1499 | 0.83 | 0.07 | 0.04 | |
| cg26002634 | ENPEP | 4 | 111396631 | 597 | 0.73 | 0.06 | 0.06 | |
| cg15810415 | ENPEP | 4 | 111397134 | 94 | 0.70 | 0.04 | 0.36 | |
| cg20773127 | ENPEP | 4 | 111397155 | 73 | 0.64 | 0.05 | 0.07 | |
| cg18944383 | ENPEP | 4 | 111397179 | 49 | 0.69 | 0.05 | 0.15 | |
| cg09248380 | ENPEP | 4 | 111397190 | 38 | 0.60 | 0.05 | 0.11 | |
| cg24192671 | ENPEP | 4 | 111397220 | 8 | 0.87 | 0.06 | 0.12 | |
| cg15406387 | ENPEP | 4 | 111397332 | 102 | 0.43 | 0.08 | 0.004 | |
| cg07587796 | ENPEP | 4 | 111397401 | 171 | 0.42 | 0.11 | 0.02 | |
| cg17854440 | ENPEP | 4 | 111397581 | 351 | 0.88 | 0.01 | 0.71 | |
| cg15711616 | ENPEP | 4 | 111397761 | 531 | 0.81 | 0.00 | 0.94 | |
| cg04748637 | ENPEP | 4 | 111397885 | 655 | 0.82 | 0.04 | 0.28 | |
| cg22888902 | ENPEP | 4 | 111398042 | 812 | 0.95 | 0.07 | 0.05 | |
| cg12913512 | CDH10 | 5 | 24488007 | 157077 | 0.89 | -0.06 | 0.15 | |
| cg17418692 | CDH10 | 5 | 24488043 | 157041 | 0.89 | -0.03 | 0.48 | |
| cg05003554 | CDH10 | 5 | 24488076 | 157008 | 0.92 | 0.02 | 0.63 | |
| cg26544752 | CDH10 | 5 | 24524850 | 120234 | 0.85 | -0.02 | 0.75 | |
| cg12559474 | CDH10 | 5 | 24622791 | 22293 | 0.92 | -0.01 | 0.77 | |
| cg02617312 | CDH10 | 5 | 24644988 | 96 | 0.07 | -0.03 | 0.47 | |
| cg26161643 | CDH10 | 5 | 24645024 | 60 | 0.09 | 0.02 | 0.57 | |
| cg05683049 | CDH10 | 5 | 24645093 | 7 | 0.13 | 0.03 | 0.52 | |
| cg14730815 | CDH10 | 5 | 24645147 | 61 | 0.06 | -0.03 | 0.34 | |
| cg01058368 | CDH10 | 5 | 24645487 | 401 | 0.87 | -0.03 | 0.45 | |
| cg01474720 | FST | 5 | 52775681 | 582 | 0.05 | 0.00 | 0.92 | |
| cg02150369 | FST | 5 | 52775702 | 561 | 0.02 | 0.00 | 0.94 | |
| cg01301664 | FST | 5 | 52775805 | 458 | 0.06 | 0.02 | 0.45 | |
| cg25645657 | FST | 5 | 52776244 | 19 | 0.07 | 0.00 | 0.97 | |
| cg05916662 | FST | 5 | 52776344 | 79 | 0.03 | 0.03 | 0.19 | |
| cg20656637 | FST | 5 | 52776630 | 365 | 0.01 | 0.01 | 0.69 | |
| cg07499072 | FST | 5 | 52776694 | 429 | 0.08 | -0.04 | 0.10 | |
| cg25404025 | FST | 5 | 52777789 | 1524 | 0.05 | 0.00 | 1.00 | |
| cg05636869 | FST | 5 | 52777880 | 1615 | 0.03 | 0.08 | 0.13 | |
| cg15902957 | FST | 5 | 52778325 | 2060 | 0.04 | -0.01 | 0.71 | |
| cg04582364 | FST | 5 | 52778441 | 2176 | 0.07 | 0.04 | 0.54 | |
| cg27372920 | FST | 5 | 52778745 | 2480 | 0.02 | -0.01 | 0.84 | |
| cg17838359 | FST | 5 | 52780901 | 4636 | 0.93 | 0.00 | 1.00 | |
| cg15911114 | FST | 5 | 52781526 | 5261 | 0.83 | 0.01 | 0.85 | |
| cg26816748 | CALD1 | 7 | 134463017 | 1146 | 0.88 | -0.08 | 0.12 | |
| cg19679250 | CALD1 | 7 | 134463072 | 1091 | 0.80 | -0.05 | 0.41 | |
| cg02382666 | CALD1 | 7 | 134463454 | 709 | 0.92 | 0.04 | 0.26 | |
| cg11425149 | CALD1 | 7 | 134463703 | 460 | 0.92 | -0.02 | 0.62 | |
| cg16253634 | CALD1 | 7 | 134464123 | 40 | 0.63 | 0.07 | 0.02 | |
| cg24956866 | CALD1 | 7 | 134464129 | 34 | 0.57 | 0.05 | 0.10 | |
| cg14009688 | CALD1 | 7 | 134464242 | 77 | 0.58 | 0.10 | 0.00 | |
| cg09337852 | CALD1 | 7 | 134464288 | 123 | 0.83 | 0.00 | 0.99 | |
| cg03362840 | CALD1 | 7 | 134464295 | 130 | 0.40 | 0.06 | 0.19 | |
| cg04874031 | CALD1 | 7 | 134464411 | 246 | 0.65 | -0.03 | 0.21 | |
| cg04738774 | CALD1 | 7 | 134473221 | 9056 | 0.93 | 0.01 | 0.81 | |
| cg18612612 | CALD1 | 7 | 134483685 | 19520 | 0.80 | -0.07 | 0.23 | |
| cg16483326 | CALD1 | 7 | 134497971 | 33806 | 0.84 | 0.03 | 0.41 | |
| cg19086309 | CALD1 | 7 | 134513289 | 49124 | 0.91 | -0.04 | 0.26 | |
| cg26990332 | CALD1 | 7 | 134516731 | 52566 | 0.89 | -0.03 | 0.70 | |
| cg15266530 | CALD1 | 7 | 134516810 | 52645 | 0.89 | 0.06 | 0.30 | |
| cg23393728 | CALD1 | 7 | 134519692 | 55527 | 0.91 | 0.00 | 0.94 | |
| cg18075666 | CALD1 | 7 | 134550430 | 25720 | 0.79 | 0.05 | 0.22 | |
| cg01043759 | CALD1 | 7 | 134564171 | 11979 | 0.89 | -0.05 | 0.33 | |
| cg27356296 | CALD1 | 7 | 134565928 | 10222 | 0.81 | 0.04 | 0.30 | |
| cg24508208 | CALD1 | 7 | 134574978 | 1172 | 0.92 | -0.08 | 0.12 | |
| cg13549461 | CALD1 | 7 | 134575145 | 1005 | 0.93 | -0.07 | 0.24 | |
| cg02516134 | CALD1 | 7 | 134575187 | 963 | 0.94 | 0.02 | 0.48 | |
| cg06390484 | CALD1 | 7 | 134575271 | 879 | 0.91 | -0.10 | 0.01 | |
| cg08698854 | CALD1 | 7 | 134575306 | 844 | 0.91 | -0.02 | 0.59 | |
| cg23190719 | CALD1 | 7 | 134575456 | 694 | 0.84 | 0.02 | 0.44 | |
| cg26915370 | CALD1 | 7 | 134575524 | 626 | 0.80 | -0.07 | 0.03 | |
| cg15905329 | CALD1 | 7 | 134575811 | 339 | 0.85 | 0.04 | 0.17 | |
| cg09916174 | CALD1 | 7 | 134576049 | 101 | 0.63 | 0.06 | 0.06 | |
| cg25390658 | CALD1 | 7 | 134576216 | 64 | 0.11 | -0.01 | 0.72 | |
| cg04655520 | CALD1 | 7 | 134576239 | 87 | 0.08 | -0.04 | 0.26 | |
| cg19488093 | CALD1 | 7 | 134576255 | 103 | 0.08 | -0.05 | 0.15 | |
| cg02087931 | CALD1 | 7 | 134576290 | 138 | 0.03 | -0.07 | 0.05 | |
| cg22480742 | CALD1 | 7 | 134576386 | 234 | 0.17 | -0.02 | 0.53 | |
| cg22369318 | CALD1 | 7 | 134585278 | 9126 | 0.91 | -0.02 | 0.44 | |
| cg17392018 | CALD1 | 7 | 134590119 | 13967 | 0.58 | 0.00 | 0.95 | |
| cg22430036 | CALD1 | 7 | 134592620 | 12610 | 0.90 | -0.07 | 0.03 | |
| cg19764295 | CALD1 | 7 | 134595401 | 9829 | 0.78 | -0.02 | 0.63 | |
| cg23461824 | CALD1 | 7 | 134605363 | 131 | 0.82 | -0.01 | 0.77 | |
| cg03032816 | CALD1 | 7 | 134613526 | 20 | 0.93 | -0.01 | 0.72 | |
| cg14519877 | CALD1 | 7 | 134617036 | 702 | 0.89 | -0.04 | 0.38 | |
| cg11253913 | CALD1 | 7 | 134617511 | 227 | 0.84 | 0.07 | 0.15 | |
| cg22328512 | CALD1 | 7 | 134620341 | 2601 | 0.94 | 0.03 | 0.41 | |
| cg07625383 | CALD1 | 7 | 134626083 | 8343 | 0.87 | -0.03 | 0.33 | |
| cg03775632 | CALD1 | 7 | 134632344 | 12380 | 0.94 | -0.03 | 0.29 | |
| cg24807761 | CALD1 | 7 | 134632531 | 12193 | 0.98 | -0.05 | 0.20 | |
| cg17231494 | CALD1 | 7 | 134633404 | 11320 | 0.91 | -0.06 | 0.22 | |
| cg09787381 | CALD1 | 7 | 134643566 | 1158 | 0.89 | -0.03 | 0.65 | |
| cg26710819 | CALD1 | 7 | 134653134 | 8408 | 0.91 | -0.06 | 0.17 | |
| cg01807619 | CRYGN | 7 | 151127947 | 2777 | 0.91 | -0.01 | 0.76 | |
| cg18560014 | CRYGN | 7 | 151129160 | 1564 | 0.98 | 0.02 | 0.59 | |
| cg08403994 | CRYGN | 7 | 151129211 | 1513 | 0.97 | 0.02 | 0.52 | |
| cg06781788 | CRYGN | 7 | 151129263 | 1461 | 0.98 | -0.04 | 0.17 | |
| cg03665494 | CRYGN | 7 | 151129313 | 1411 | 0.92 | 0.03 | 0.35 | |
| cg18810691 | CRYGN | 7 | 151130479 | 245 | 0.46 | 0.04 | 0.09 | |
| cg24929514 | CRYGN | 7 | 151132764 | 646 | 0.86 | 0.04 | 0.11 | |
| cg24449445 | CRYGN | 7 | 151134527 | 1115 | 0.94 | -0.02 | 0.46 | |
| cg06288696 | CRYGN | 7 | 151137008 | 90 | 0.04 | -0.04 | 0.13 | |
| cg02512352 | CRYGN | 7 | 151137048 | 50 | 0.02 | -0.04 | 0.14 | |
| cg23273694 | CRYGN | 7 | 151137162 | 62 | 0.03 | -0.03 | 0.40 | |
| cg05551889 | CRYGN | 7 | 151137192 | 92 | 0.04 | 0.01 | 0.86 | |
| cg22830895 | CRYGN | 7 | 151137476 | 376 | 0.43 | 0.08 | 0.003 | |
| cg16044810 | CRYGN | 7 | 151137746 | 152 | 0.80 | -0.05 | 0.08 | |
| cg23666844 | CRYGN | 7 | 151137882 | 16 | 0.55 | -0.01 | 0.79 | |
| cg21171320 | TRPM3 | 9 | 73178360 | 148786 | 0.32 | -0.09 | 0.02 | |
| cg13675278 | TRPM3 | 9 | 73189590 | 160016 | 0.91 | -0.01 | 0.68 | |
| cg14265537 | TRPM3 | 9 | 73404674 | 20325 | 0.87 | 0.02 | 0.71 | |
| cg02610438 | TRPM3 | 9 | 73424620 | 379 | 0.91 | 0.01 | 0.75 | |
| cg21251785 | TRPM3 | 9 | 73484407 | 432 | 0.89 | -0.04 | 0.37 | |
| cg14165911 | TRPM3 | 9 | 73594877 | 110902 | 0.77 | 0.06 | 0.27 | |
| cg16832407 | TRPM3 | 9 | 73736539 | 24 | 0.28 | 0.00 | 0.93 | |
| cg21249093 | TRPM3 | 9 | 73737300 | 785 | 0.69 | 0.05 | 0.28 | |
| cg04725234 | TMEM38B | 9 | 108456639 | 166 | 0.02 | -0.01 | 0.59 | |
| cg00556719 | TMEM38B | 9 | 108457484 | 677 | 0.08 | 0.00 | 0.82 | |
| cg14519696 | TMEM38B | 9 | 108459452 | 2645 | 0.90 | -0.02 | 0.54 | |
| cg13459284 | FRMD4A | 10 | 13688089 | 7145 | 0.94 | 0.01 | 0.87 | |
| cg06822062 | FRMD4A | 10 | 13688106 | 7162 | 0.95 | 0.00 | 0.95 | |
| cg24008634 | FRMD4A | 10 | 13694895 | 13951 | 0.94 | 0.04 | 0.34 | |
| cg05583456 | FRMD4A | 10 | 13698485 | 17541 | 0.90 | 0.01 | 0.80 | |
| cg04174358 | FRMD4A | 10 | 13698563 | 17619 | 0.88 | 0.01 | 0.69 | |
| cg01164291 | FRMD4A | 10 | 13699426 | 18482 | 0.83 | -0.03 | 0.19 | |
| cg09684112 | FRMD4A | 10 | 13701447 | 20503 | 0.18 | -0.09 | 0.11 | |
| cg26477221 | FRMD4A | 10 | 13702163 | 21219 | 0.04 | 0.04 | 0.24 | |
| cg13734953 | FRMD4A | 10 | 13708059 | 27115 | 0.97 | -0.03 | 0.39 | |
| cg23731992 | FRMD4A | 10 | 13708083 | 27139 | 0.98 | 0.04 | 0.14 | |
| cg14702215 | FRMD4A | 10 | 13708110 | 27166 | 0.91 | 0.02 | 0.59 | |
| cg00246402 | FRMD4A | 10 | 13708238 | 27294 | 0.92 | -0.03 | 0.48 | |
| cg11197893 | FRMD4A | 10 | 13708261 | 27317 | 0.92 | -0.05 | 0.17 | |
| cg26334209 | FRMD4A | 10 | 13726634 | 45690 | 0.90 | 0.01 | 0.84 | |
| cg18698819 | FRMD4A | 10 | 13736003 | 55059 | 0.92 | -0.06 | 0.21 | |
| cg15658978 | FRMD4A | 10 | 13736020 | 55076 | 0.83 | 0.00 | 0.92 | |
| cg15777335 | FRMD4A | 10 | 13736109 | 55165 | 0.98 | 0.03 | 0.41 | |
| cg05335944 | FRMD4A | 10 | 13749010 | 68066 | 0.92 | -0.03 | 0.42 | |
| cg22923409 | FRMD4A | 10 | 13749245 | 68301 | 0.13 | -0.04 | 0.22 | |
| cg19120064 | FRMD4A | 10 | 13749285 | 68341 | 0.06 | 0.01 | 0.82 | |
| cg00406392 | FRMD4A | 10 | 13749405 | 68461 | 0.04 | -0.02 | 0.63 | |
| cg04055819 | FRMD4A | 10 | 13749602 | 68658 | 0.04 | 0.00 | 0.87 | |
| cg20517614 | FRMD4A | 10 | 13749625 | 68681 | 0.03 | -0.06 | 0.02 | |
| cg24951100 | FRMD4A | 10 | 13760075 | 79131 | 0.08 | -0.01 | 0.82 | |
| cg02859866 | FRMD4A | 10 | 13760165 | 79221 | 0.10 | 0.01 | 0.53 | |
| cg19805033 | FRMD4A | 10 | 13766768 | 85824 | 0.86 | 0.05 | 0.18 | |
| cg20152304 | FRMD4A | 10 | 13771107 | 90163 | 0.93 | -0.01 | 0.71 | |
| cg13473894 | FRMD4A | 10 | 13771383 | 90439 | 0.98 | -0.04 | 0.12 | |
| cg12656653 | FRMD4A | 10 | 13771427 | 90483 | 0.97 | -0.02 | 0.53 | |
| cg23873415 | FRMD4A | 10 | 13771465 | 90521 | 0.98 | 0.00 | 0.92 | |
| cg09309979 | FRMD4A | 10 | 13818133 | 137189 | 0.82 | 0.03 | 0.41 | |
| cg03775372 | FRMD4A | 10 | 13830045 | 149101 | 0.76 | -0.03 | 0.09 | |
| cg08791347 | FRMD4A | 10 | 13831250 | 150306 | 0.36 | 0.03 | 0.24 | |
| cg10528559 | FRMD4A | 10 | 13836757 | 155813 | 0.91 | -0.03 | 0.33 | |
| cg11703729 | FRMD4A | 10 | 13851615 | 162753 | 0.91 | 0.09 | 0.02 | |
| cg25932446 | FRMD4A | 10 | 13858592 | 155776 | 0.89 | -0.10 | 0.02 | |
| cg10822352 | FRMD4A | 10 | 13876378 | 137990 | 0.87 | -0.02 | 0.29 | |
| cg00593298 | FRMD4A | 10 | 13890043 | 124325 | 0.49 | -0.06 | 0.10 | |
| cg03783391 | FRMD4A | 10 | 13908414 | 105954 | 0.89 | -0.03 | 0.52 | |
| cg05392293 | FRMD4A | 10 | 13911331 | 103037 | 0.73 | 0.07 | 0.16 | |
| cg22959742 | FRMD4A | 10 | 13913931 | 100437 | 0.55 | 0.00 | 0.93 | |
| cg19648751 | FRMD4A | 10 | 13923824 | 90544 | 0.68 | 0.02 | 0.48 | |
| cg03493513 | FRMD4A | 10 | 13925050 | 89318 | 0.62 | 0.04 | 0.13 | |
| cg14672137 | FRMD4A | 10 | 13931052 | 83316 | 0.91 | -0.01 | 0.82 | |
| cg02347573 | FRMD4A | 10 | 13931592 | 82776 | 0.93 | 0.00 | 0.88 | |
| cg06879226 | FRMD4A | 10 | 13932842 | 81526 | 0.06 | -0.03 | 0.50 | |
| cg15803756 | FRMD4A | 10 | 13932894 | 81474 | 0.09 | -0.05 | 0.21 | |
| cg25453664 | FRMD4A | 10 | 13933021 | 81347 | 0.08 | 0.08 | 0.06 | |
| cg22673543 | FRMD4A | 10 | 13933229 | 81139 | 0.51 | 0.02 | 0.36 | |
| cg00296121 | FRMD4A | 10 | 13933247 | 81121 | 0.07 | -0.03 | 0.39 | |
| cg02451730 | FRMD4A | 10 | 13933452 | 80916 | 0.03 | 0.00 | 0.90 | |
| cg07071978 | FRMD4A | 10 | 13933652 | 80716 | 0.05 | 0.04 | 0.58 | |
| cg14649650 | FRMD4A | 10 | 13933996 | 80372 | 0.02 | 0.02 | 0.47 | |
| cg20130213 | FRMD4A | 10 | 13934169 | 80199 | 0.09 | -0.07 | 0.02 | |
| cg04557357 | FRMD4A | 10 | 13934255 | 80113 | 0.13 | 0.01 | 0.89 | |
| cg07315858 | FRMD4A | 10 | 13934368 | 80000 | 0.17 | -0.01 | 0.65 | |
| cg19536508 | FRMD4A | 10 | 13936296 | 78072 | 0.94 | -0.01 | 0.84 | |
| cg24876648 | FRMD4A | 10 | 13965645 | 48723 | 0.91 | 0.01 | 0.77 | |
| cg07629776 | FRMD4A | 10 | 13972210 | 42158 | 0.59 | 0.23 | 0.03 | |
| cg23556574 | FRMD4A | 10 | 13981154 | 33214 | 0.85 | -0.03 | 0.47 | |
| cg23443098 | FRMD4A | 10 | 13987636 | 26732 | 0.80 | 0.02 | 0.57 | |
| cg27572370 | FRMD4A | 10 | 14002394 | 11974 | 0.57 | 0.06 | 0.42 | |
| cg20179726 | FRMD4A | 10 | 14014419 | 49 | 0.88 | 0.11 | 0.01 | |
| cg04819048 | FRMD4A | 10 | 14014548 | 178 | 0.94 | -0.05 | 0.23 | |
| cg05163663 | FRMD4A | 10 | 14014553 | 183 | 0.91 | -0.01 | 0.88 | |
| cg00551146 | FRMD4A | 10 | 14014579 | 209 | 0.75 | -0.02 | 0.65 | |
| cg01267150 | FRMD4A | 10 | 14014651 | 281 | 0.45 | 0.03 | 0.55 | |
| cg20174247 | FRMD4A | 10 | 14032690 | 17469 | 0.92 | 0.02 | 0.59 | |
| cg23011788 | FRMD4A | 10 | 14050305 | 144 | 0.08 | 0.09 | 0.05 | |
| cg16241033 | FRMD4A | 10 | 14050455 | 294 | 0.10 | -0.01 | 0.80 | |
| cg05888872 | FRMD4A | 10 | 14050479 | 318 | 0.17 | -0.12 | 0.09 | |
| cg02101203 | FRMD4A | 10 | 14050521 | 360 | 0.12 | -0.10 | 0.11 | |
| cg26984593 | FRMD4A | 10 | 14051636 | 1475 | 0.47 | -0.06 | 0.13 | |
| cg08734931 | FRMD4A | 10 | 14051649 | 1488 | 0.45 | -0.10 | 0.04 | |
| cg02284273 | FRMD4A | 10 | 14051679 | 1518 | 0.55 | -0.04 | 0.42 | |
| cg03179435 | FRMD4A | 10 | 14051821 | 1660 | 0.31 | -0.16 | 0.03 | |
| cg19998150 | FRMD4A | 10 | 14051838 | 1677 | 0.16 | -0.24 | 0.01 | |
| cg21371809 | FRMD4A | 10 | 14052028 | 1867 | 0.31 | -0.08 | 0.12 | |
| cg25344406 | FRMD4A | 10 | 14065229 | 15068 | 0.94 | 0.03 | 0.36 | |
| cg07828294 | FRMD4A | 10 | 14091532 | 41371 | 0.93 | 0.02 | 0.57 | |
| cg06858087 | FRMD4A | 10 | 14099154 | 48993 | 0.93 | -0.08 | 0.09 | |
| cg15470075 | FRMD4A | 10 | 14105835 | 55674 | 0.91 | 0.05 | 0.13 | |
| cg17920246 | FRMD4A | 10 | 14116424 | 66263 | 0.89 | -0.01 | 0.74 | |
| cg16634545 | FRMD4A | 10 | 14129104 | 78943 | 0.78 | -0.09 | 0.22 | |
| cg25045228 | FRMD4A | 10 | 14146672 | 96511 | 0.79 | 0.02 | 0.56 | |
| cg06339003 | FRMD4A | 10 | 14188049 | 137888 | 0.93 | -0.04 | 0.26 | |
| cg04941706 | FRMD4A | 10 | 14215314 | 157551 | 0.63 | 0.08 | 0.04 | |
| cg06035247 | FRMD4A | 10 | 14215708 | 157157 | 0.04 | 0.01 | 0.78 | |
| cg09650487 | FRMD4A | 10 | 14215771 | 157094 | 0.05 | 0.04 | 0.27 | |
| cg07668267 | FRMD4A | 10 | 14216014 | 156851 | 0.05 | -0.02 | 0.71 | |
| cg18909530 | FRMD4A | 10 | 14216100 | 156765 | 0.09 | 0.02 | 0.15 | |
| cg00407040 | FRMD4A | 10 | 14247935 | 124930 | 0.93 | -0.01 | 0.72 | |
| cg23037642 | FRMD4A | 10 | 14282687 | 90178 | 0.80 | -0.05 | 0.33 | |
| cg03355808 | FRMD4A | 10 | 14302678 | 70187 | 0.74 | 0.02 | 0.51 | |
| cg20381271 | FRMD4A | 10 | 14320625 | 52240 | 0.82 | 0.03 | 0.39 | |
| cg20344448 | FRMD4A | 10 | 14372431 | 434 | 0.76 | 0.10 | 0.004 | |
| cg05755354 | FRMD4A | 10 | 14372596 | 269 | 0.88 | 0.00 | 0.90 | |
| cg11813497 | FRMD4A | 10 | 14372879 | 12 | 0.81 | 0.14 | 0.002 | |
| cg25464840 | FRMD4A | 10 | 14372910 | 43 | 0.68 | 0.10 | 0.001 | |
| cg15507334 | FRMD4A | 10 | 14372913 | 46 | 0.56 | 0.10 | 0.0001 | |
| cg05018023 | FRMD4A | 10 | 14374096 | 1229 | 0.88 | -0.04 | 0.32 | |
| cg06395652 | PCDH15 | 10 | 55568997 | 855052 | 0.85 | 0.01 | 0.78 | |
| cg25035332 | PCDH15 | 10 | 55587268 | 836781 | 0.87 | -0.06 | 0.44 | |
| cg11571884 | PCDH15 | 10 | 55685173 | 738876 | 0.86 | -0.10 | 0.03 | |
| cg02224394 | PCDH15 | 10 | 55750757 | 673292 | 0.98 | -0.07 | 0.02 | |
| cg03808835 | PCDH15 | 10 | 56560945 | 105 | 0.08 | -0.06 | 0.10 | |
| cg20588045 | PCDH15 | 10 | 56561096 | 44 | 0.30 | 0.04 | 0.24 | |
| cg25592910 | PCDH15 | 10 | 56561124 | 72 | 0.52 | 0.03 | 0.24 | |
| cg02613108 | PCDH15 | 10 | 56561134 | 82 | 0.36 | 0.00 | 0.96 | |
| cg24021130 | PCDH15 | 10 | 56561213 | 161 | 0.58 | 0.05 | 0.23 | |
| cg27329559 | PCDH15 | 10 | 56561543 | 491 | 0.60 | 0.08 | 0.04 | |
| cg03088705 | XPNPEP1 | 10 | 111624928 | 19101 | 0.89 | -0.03 | 0.41 | |
| cg04350202 | XPNPEP1 | 10 | 111653363 | 9332 | 0.47 | -0.05 | 0.11 | |
| cg19777638 | XPNPEP1 | 10 | 111655132 | 11101 | 0.95 | -0.10 | 0.002 | |
| cg18780288 | XPNPEP1 | 10 | 111659903 | 15872 | 0.26 | -0.07 | 0.13 | |
| cg00817464 | XPNPEP1 | 10 | 111662876 | 18845 | 0.78 | 0.17 | 0.08 | |
| cg09352789 | XPNPEP1 | 10 | 111682693 | 617 | 0.29 | 0.00 | 0.88 | |
| cg09688285 | XPNPEP1 | 10 | 111682808 | 502 | 0.03 | 0.07 | 0.01 | |
| cg06459669 | XPNPEP1 | 10 | 111683143 | 167 | 0.09 | -0.01 | 0.89 | |
| cg11055926 | XPNPEP1 | 10 | 111683227 | 83 | 0.31 | 0.02 | 0.29 | |
| cg15635851 | XPNPEP1 | 10 | 111683342 | 30 | 0.02 | -0.03 | 0.09 | |
| cg05697697 | XPNPEP1 | 10 | 111683345 | 33 | 0.02 | 0.02 | 0.40 | |
| cg09292873 | XPNPEP1 | 10 | 111683347 | 35 | 0.12 | -0.01 | 0.84 | |
| cg11150068 | XPNPEP1 | 10 | 111683422 | 110 | 0.05 | 0.01 | 0.61 | |
| cg17288102 | XPNPEP1 | 10 | 111683471 | 159 | 0.04 | -0.02 | 0.66 | |
| cg18060846 | XPNPEP1 | 10 | 111683482 | 170 | 0.03 | -0.01 | 0.56 | |
| cg06688014 | XPNPEP1 | 10 | 111683554 | 242 | 0.32 | 0.00 | 0.96 | |
| cg17201651 | XPNPEP1 | 10 | 111683631 | 319 | 0.53 | -0.08 | 0.08 | |
| cg17093267 | XPNPEP1 | 10 | 111683654 | 342 | 0.53 | -0.01 | 0.74 | |
| cg19766102 | XPNPEP1 | 10 | 111683738 | 426 | 0.92 | 0.04 | 0.27 | |
| cg18087943 | IGF2AS | 11 | 2160540 | 335 | 0.02 | 0.00 | 0.97 | |
| cg14608156 | IGF2AS | 11 | 2160554 | 349 | 0.03 | 0.03 | 0.21 | |
| cg12773325 | IGF2AS | 11 | 2160560 | 355 | 0.03 | 0.04 | 0.16 | |
| cg12614029 | IGF2AS | 11 | 2160564 | 359 | 0.03 | 0.00 | 0.88 | |
| cg08162473 | IGF2AS | 11 | 2160721 | 516 | 0.02 | -0.04 | 0.16 | |
| cg03760951 | IGF2AS | 11 | 2160875 | 670 | 0.10 | 0.01 | 0.90 | |
| cg20339650 | IGF2AS | 11 | 2160882 | 677 | 0.16 | 0.01 | 0.46 | |
| cg19002337 | IGF2AS | 11 | 2160904 | 699 | 0.03 | -0.04 | 0.18 | |
| cg24366657 | IGF2AS | 11 | 2160932 | 727 | 0.05 | -0.01 | 0.81 | |
| cg24917382 | IGF2AS | 11 | 2160953 | 748 | 0.15 | 0.04 | 0.07 | |
| cg16415340 | IGF2AS | 11 | 2160964 | 759 | 0.04 | -0.06 | 0.12 | |
| cg21667878 | IGF2AS | 11 | 2160980 | 775 | 0.03 | -0.05 | 0.25 | |
| cg11915650 | IGF2AS | 11 | 2161009 | 748 | 0.05 | -0.04 | 0.37 | |
| cg17434309 | IGF2AS | 11 | 2161079 | 678 | 0.07 | 0.01 | 0.65 | |
| cg05859777 | IGF2AS | 11 | 2161102 | 655 | 0.08 | 0.00 | 0.92 | |
| cg01667319 | IGF2AS | 11 | 2161128 | 629 | 0.07 | 0.00 | 0.93 | |
| cg05452899 | IGF2AS | 11 | 2161133 | 624 | 0.09 | -0.02 | 0.59 | |
| cg10037494 | IGF2AS | 11 | 2161135 | 622 | 0.03 | -0.03 | 0.27 | |
| cg26517849 | IGF2AS | 11 | 2161141 | 616 | 0.03 | 0.00 | 0.87 | |
| cg17037101 | IGF2AS | 11 | 2161314 | 443 | 0.05 | -0.03 | 0.47 | |
| cg09694722 | IGF2AS | 11 | 2161318 | 439 | 0.11 | 0.00 | 0.96 | |
| cg19371526 | IGF2AS | 11 | 2161341 | 416 | 0.10 | 0.01 | 0.86 | |
| cg23676551 | IGF2AS | 11 | 2161374 | 383 | 0.10 | -0.03 | 0.42 | |
| cg15393937 | IGF2AS | 11 | 2161383 | 374 | 0.12 | -0.04 | 0.32 | |
| cg23905216 | IGF2AS | 11 | 2161390 | 367 | 0.18 | 0.04 | 0.09 | |
| cg19443075 | IGF2AS | 11 | 2161403 | 354 | 0.17 | 0.04 | 0.23 | |
| cg22287492 | IGF2AS | 11 | 2161411 | 346 | 0.18 | 0.01 | 0.63 | |
| cg10659464 | IGF2AS | 11 | 2161445 | 312 | 0.18 | 0.01 | 0.72 | |
| cg02835822 | IGF2AS | 11 | 2161456 | 301 | 0.13 | -0.04 | 0.15 | |
| cg13756879 | IGF2AS | 11 | 2161473 | 284 | 0.05 | 0.03 | 0.43 | |
| cg14188639 | IGF2AS | 11 | 2161544 | 213 | 0.14 | 0.09 | 0.03 | |
| cg25163476 | IGF2AS | 11 | 2161586 | 171 | 0.09 | -0.01 | 0.81 | |
| cg13791131 | IGF2AS | 11 | 2161892 | 133 | 0.09 | 0.00 | 0.96 | |
| cg25574024 | IGF2AS | 11 | 2161894 | 135 | 0.09 | 0.01 | 0.78 | |
| cg23030069 | IGF2AS | 11 | 2162183 | 157 | 0.04 | 0.02 | 0.44 | |
| cg06460568 | IGF2AS | 11 | 2162211 | 129 | 0.03 | -0.07 | 0.10 | |
| cg24047810 | IGF2AS | 11 | 2162341 | 0 | 0.18 | -0.03 | 0.24 | |
| cg17462140 | IGF2AS | 11 | 2162363 | 21 | 0.13 | -0.01 | 0.45 | |
| cg24431667 | IGF2AS | 11 | 2162387 | 45 | 0.06 | -0.03 | 0.41 | |
| cg05444816 | IGF2AS | 11 | 2162406 | 64 | 0.09 | -0.02 | 0.50 | |
| cg15508379 | IGF2AS | 11 | 2162438 | 96 | 0.12 | 0.02 | 0.54 | |
| cg13928782 | IGF2AS | 11 | 2162445 | 103 | 0.08 | -0.01 | 0.80 | |
| cg17300736 | IGF2AS | 11 | 2162475 | 133 | 0.05 | 0.04 | 0.31 | |
| cg11701022 | IGF2AS | 11 | 2162478 | 136 | 0.23 | 0.02 | 0.34 | |
| cg01368777 | IGF2AS | 11 | 2162483 | 141 | 0.17 | 0.04 | 0.05 | |
| cg21237591 | IGF2AS | 11 | 2162510 | 168 | 0.17 | 0.02 | 0.55 | |
| cg22225943 | IGF2AS | 11 | 2162536 | 194 | 0.18 | 0.03 | 0.40 | |
| cg08014499 | IGF2AS | 11 | 2162545 | 203 | 0.20 | 0.00 | 0.98 | |
| cg05203776 | IGF2AS | 11 | 2162582 | 240 | 0.17 | 0.03 | 0.20 | |
| cg20088847 | IGF2AS | 11 | 2162616 | 274 | 0.09 | 0.04 | 0.25 | |
| cg15168906 | IGF2AS | 11 | 2162783 | 441 | 0.58 | 0.02 | 0.67 | |
| cg19131227 | IGF2AS | 11 | 2162917 | 575 | 0.31 | 0.05 | 0.12 | |
| cg24781163 | IGF2AS | 11 | 2162930 | 588 | 0.57 | 0.01 | 0.83 | |
| cg05323345 | IGF2AS | 11 | 2163174 | 832 | 0.27 | 0.05 | 0.31 | |
| cg26719629 | IGF2AS | 11 | 2163299 | 957 | 0.35 | 0.04 | 0.44 | |
| cg14895961 | IGF2AS | 11 | 2163513 | 1171 | 0.30 | 0.01 | 0.74 | |
| cg05777976 | IGF2AS | 11 | 2163538 | 1196 | 0.07 | -0.02 | 0.60 | |
| cg02425416 | IGF2AS | 11 | 2163808 | 1466 | 0.22 | 0.07 | 0.10 | |
| cg12877935 | IGF2AS | 11 | 2164780 | 2438 | 0.03 | 0.05 | 0.08 | |
| cg10501065 | IGF2AS | 11 | 2164976 | 2634 | 0.11 | 0.04 | 0.27 | |
| cg04112019 | IGF2AS | 11 | 2165136 | 2794 | 0.07 | -0.06 | 0.09 | |
| cg20792294 | IGF2AS | 11 | 2165425 | 3083 | 0.03 | -0.01 | 0.87 | |
| cg21532432 | IGF2AS | 11 | 2165434 | 3092 | 0.04 | -0.06 | 0.20 | |
| cg12322132 | IGF2AS | 11 | 2165656 | 3314 | 0.06 | 0.07 | 0.04 | |
| cg16817891 | IGF2AS | 11 | 2165827 | 3485 | 0.05 | -0.02 | 0.68 | |
| cg11005826 | IGF2AS | 11 | 2165961 | 3619 | 0.13 | -0.02 | 0.64 | |
| cg19642877 | IGF2AS | 11 | 2168625 | 2207 | 0.54 | 0.02 | 0.20 | |
| cg13473383 | ZDHHC5 | 11 | 57434378 | 1095 | 0.16 | -0.03 | 0.30 | |
| cg26816128 | ZDHHC5 | 11 | 57434555 | 918 | 0.05 | 0.00 | 0.91 | |
| cg05995172 | ZDHHC5 | 11 | 57434667 | 806 | 0.04 | -0.05 | 0.25 | |
| cg00251705 | ZDHHC5 | 11 | 57434686 | 787 | 0.03 | -0.05 | 0.03 | |
| cg21606915 | ZDHHC5 | 11 | 57434710 | 763 | 0.04 | -0.01 | 0.70 | |
| cg10284592 | ZDHHC5 | 11 | 57434942 | 531 | 0.07 | -0.02 | 0.54 | |
| cg18039855 | ZDHHC5 | 11 | 57434952 | 521 | 0.07 | 0.01 | 0.67 | |
| cg22663124 | ZDHHC5 | 11 | 57435473 | 0 | 0.04 | 0.03 | 0.37 | |
| cg18493899 | ZDHHC5 | 11 | 57435499 | 24 | 0.02 | 0.01 | 0.35 | |
| cg06339248 | ZDHHC5 | 11 | 57435803 | 328 | 0.11 | -0.01 | 0.86 | |
| cg17966709 | ZDHHC5 | 11 | 57435952 | 477 | 0.05 | 0.03 | 0.48 | |
| cg09122158 | ZDHHC5 | 11 | 57436238 | 763 | 0.06 | 0.07 | 0.12 | |
| cg13582500 | ZDHHC5 | 11 | 57436966 | 1491 | 0.85 | -0.05 | 0.25 | |
| cg10363915 | ZDHHC5 | 11 | 57438680 | 3205 | 0.94 | -0.04 | 0.32 | |
| cg14596450 | ZDHHC5 | 11 | 57459649 | 10697 | 0.88 | -0.03 | 0.33 | |
| cg08140255 | ZDHHC5 | 11 | 57467618 | 12054 | 0.95 | -0.01 | 0.75 | |
| cg13745279 | C11orf52 | 11 | 111788810 | 5349 | 0.90 | 0.03 | 0.39 | |
| cg19053142 | C11orf52 | 11 | 111789132 | 5671 | 0.90 | -0.05 | 0.25 | |
| cg23921031 | C11orf52 | 11 | 111789452 | 5991 | 0.92 | -0.02 | 0.64 | |
| cg11845417 | C11orf52 | 11 | 111789613 | 6152 | 0.63 | 0.07 | 0.003 | |
| cg05697249 | C11orf52 | 11 | 111789693 | 6232 | 0.70 | 0.09 | 0.004 | |
| cg15318568 | C11orf52 | 11 | 111794346 | 3521 | 0.80 | -0.06 | 0.20 | |
| cg12247500 | C11orf52 | 11 | 111796862 | 1005 | 0.98 | -0.03 | 0.26 | |
| cg10055139 | C11orf52 | 11 | 111797552 | 315 | 0.03 | 0.00 | 0.91 | |
| cg09327582 | BAZ1A | 14 | 35236912 | 52882 | 0.94 | -0.03 | 0.54 | |
| cg19530972 | BAZ1A | 14 | 35246575 | 62545 | 0.94 | -0.05 | 0.35 | |
| cg18525312 | BAZ1A | 14 | 35312638 | 31499 | 0.85 | 0.05 | 0.20 | |
| cg01831476 | BAZ1A | 14 | 35342618 | 1519 | 0.03 | 0.00 | 0.95 | |
| cg23512654 | BAZ1A | 14 | 35343213 | 924 | 0.02 | 0.05 | 0.11 | |
| cg16746455 | BAZ1A | 14 | 35343581 | 556 | 0.04 | -0.02 | 0.43 | |
| cg06089712 | BAZ1A | 14 | 35343862 | 275 | 0.04 | -0.01 | 0.82 | |
| cg23868672 | BAZ1A | 14 | 35344258 | 119 | 0.02 | 0.01 | 0.70 | |
| cg23422263 | BAZ1A | 14 | 35344518 | 334 | 0.04 | -0.01 | 0.82 | |
| cg07414384 | BAZ1A | 14 | 35344667 | 185 | 0.06 | 0.06 | 0.18 | |
| cg05294307 | BAZ1A | 14 | 35346193 | 1339 | 0.91 | -0.05 | 0.32 | |
| cg26967526 | BAZ1A | 14 | 35346199 | 1345 | 0.90 | 0.03 | 0.45 | |
| cg08937732 | SMPD3 | 16 | 68393121 | 7137 | 0.98 | 0.00 | 0.93 | |
| cg26906642 | SMPD3 | 16 | 68394729 | 8745 | 0.87 | -0.01 | 0.65 | |
| cg05392527 | SMPD3 | 16 | 68394825 | 8841 | 0.81 | -0.04 | 0.13 | |
| cg02226672 | SMPD3 | 16 | 68398533 | 7756 | 0.50 | -0.01 | 0.76 | |
| cg10422047 | SMPD3 | 16 | 68401557 | 4732 | 0.92 | 0.01 | 0.80 | |
| cg16658734 | SMPD3 | 16 | 68401882 | 4407 | 0.87 | -0.03 | 0.21 | |
| cg26899718 | SMPD3 | 16 | 68404797 | 1492 | 0.69 | 0.00 | 0.86 | |
| cg03599928 | SMPD3 | 16 | 68405168 | 1121 | 0.77 | 0.04 | 0.18 | |
| cg09578614 | SMPD3 | 16 | 68405406 | 883 | 0.99 | 0.00 | 0.78 | |
| cg01210622 | SMPD3 | 16 | 68405731 | 558 | 0.94 | -0.01 | 0.76 | |
| cg04747382 | SMPD3 | 16 | 68406461 | 170 | 0.95 | -0.02 | 0.47 | |
| cg04713743 | SMPD3 | 16 | 68406468 | 177 | 0.92 | 0.02 | 0.61 | |
| cg03873826 | SMPD3 | 16 | 68407311 | 1020 | 0.84 | -0.01 | 0.71 | |
| cg27045405 | SMPD3 | 16 | 68409791 | 3500 | 0.91 | -0.04 | 0.17 | |
| cg07735969 | SMPD3 | 16 | 68418473 | 12182 | 0.42 | 0.01 | 0.55 | |
| cg04685738 | SMPD3 | 16 | 68421884 | 15593 | 0.85 | 0.10 | 0.001 | |
| cg02593381 | SMPD3 | 16 | 68434111 | 27820 | 0.49 | 0.04 | 0.05 | |
| cg01041405 | SMPD3 | 16 | 68440620 | 34329 | 0.93 | 0.00 | 0.93 | |
| cg05591728 | SMPD3 | 16 | 68477022 | 5386 | 0.81 | -0.03 | 0.35 | |
| cg05430886 | SMPD3 | 16 | 68479922 | 2486 | 0.92 | -0.02 | 0.68 | |
| cg08890345 | SMPD3 | 16 | 68481248 | 1160 | 0.29 | 0.05 | 0.20 | |
| cg05948940 | SMPD3 | 16 | 68481342 | 1066 | 0.11 | 0.01 | 0.91 | |
| cg00891541 | SMPD3 | 16 | 68481487 | 921 | 0.59 | -0.02 | 0.62 | |
| cg10556064 | SMPD3 | 16 | 68481489 | 919 | 0.60 | -0.01 | 0.78 | |
| cg05144928 | SMPD3 | 16 | 68481543 | 865 | 0.47 | 0.10 | 0.03 | |
| cg12136772 | SMPD3 | 16 | 68481813 | 595 | 0.34 | 0.03 | 0.26 | |
| cg22116290 | SMPD3 | 16 | 68481968 | 440 | 0.11 | 0.00 | 0.94 | |
| cg05676789 | SMPD3 | 16 | 68482144 | 264 | 0.05 | -0.03 | 0.52 | |
| cg23758485 | SMPD3 | 16 | 68482202 | 206 | 0.02 | 0.00 | 0.85 | |
| cg16694003 | SMPD3 | 16 | 68482277 | 131 | 0.02 | -0.01 | 0.71 | |
| cg15201635 | SMPD3 | 16 | 68482637 | 227 | 0.06 | 0.04 | 0.22 | |
| cg09202227 | SMPD3 | 16 | 68482715 | 305 | 0.05 | -0.01 | 0.89 | |
| cg07461772 | SMPD3 | 16 | 68482809 | 399 | 0.10 | 0.09 | 0.09 | |
| cg10426893 | SMPD3 | 16 | 68482821 | 411 | 0.22 | 0.02 | 0.47 | |
| cg06106242 | SMPD3 | 16 | 68482941 | 531 | 0.59 | 0.00 | 0.96 | |
| cg19297232 | SMPD3 | 16 | 68483021 | 611 | 0.76 | 0.02 | 0.74 | |
| cg17217677 | SMPD3 | 16 | 68483194 | 784 | 0.83 | 0.05 | 0.10 | |
| cg17942096 | RFXANK | 19 | 19301711 | 1296 | 0.87 | -0.01 | 0.73 | |
| cg14580737 | RFXANK | 19 | 19301780 | 1227 | 0.86 | -0.02 | 0.59 | |
| cg15715094 | RFXANK | 19 | 19302729 | 278 | 0.03 | -0.02 | 0.53 | |
| cg02178774 | RFXANK | 19 | 19302803 | 204 | 0.06 | 0.07 | 0.05 | |
| cg18151074 | RFXANK | 19 | 19302985 | 22 | 0.02 | 0.01 | 0.75 | |
| cg24922631 | RFXANK | 19 | 19303269 | 130 | 0.02 | 0.03 | 0.56 | |
| cg09421020 | RFXANK | 19 | 19303389 | 10 | 0.07 | -0.02 | 0.48 | |
| cg16196853 | RFXANK | 19 | 19303443 | 42 | 0.04 | -0.03 | 0.31 | |
| cg12208283 | RFXANK | 19 | 19303500 | 99 | 0.07 | 0.05 | 0.11 | |
| cg03218003 | RFXANK | 19 | 19303504 | 103 | 0.02 | 0.02 | 0.29 | |
| cg15108830 | RFXANK | 19 | 19303541 | 140 | 0.09 | 0.03 | 0.35 | |
| cg23926253 | RFXANK | 19 | 19303735 | 20 | 0.02 | 0.04 | 0.02 | |
| cg08878360 | RFXANK | 19 | 19303979 | 222 | 0.04 | 0.07 | 0.02 | |
| cg00495352 | RFXANK | 19 | 19304836 | 87 | 0.86 | -0.01 | 0.70 | |
| cg06446466 | RFXANK | 19 | 19310798 | 2051 | 0.95 | 0.02 | 0.55 | |
| cg09143663 | BACH1 | 21 | 30670416 | 803 | 0.75 | -0.02 | 0.64 | |
| cg18767088 | BACH1 | 21 | 30670919 | 300 | 0.07 | 0.07 | 0.00 | |
| cg10106363 | BACH1 | 21 | 30670923 | 296 | 0.01 | 0.02 | 0.29 | |
| cg24788122 | BACH1 | 21 | 30670943 | 276 | 0.02 | -0.03 | 0.07 | |
| cg26921846 | BACH1 | 21 | 30671131 | 88 | 0.02 | -0.04 | 0.09 | |
| cg12008680 | BACH1 | 21 | 30671144 | 75 | 0.02 | 0.03 | 0.34 | |
| cg15572535 | BACH1 | 21 | 30671155 | 64 | 0.02 | -0.03 | 0.06 | |
| cg03693762 | BACH1 | 21 | 30671158 | 61 | 0.01 | 0.04 | 0.00 | |
| cg05699136 | BACH1 | 21 | 30671375 | 154 | 0.02 | 0.01 | 0.59 | |
| cg07434500 | BACH1 | 21 | 30671675 | 61 | 0.02 | 0.05 | 0.02 | |
| cg25531249 | BACH1 | 21 | 30671681 | 55 | 0.02 | -0.01 | 0.50 | |
| cg19199142 | BACH1 | 21 | 30671683 | 53 | 0.01 | 0.02 | 0.11 | |
| cg14918353 | BACH1 | 21 | 30671695 | 41 | 0.04 | -0.01 | 0.79 | |
| cg26680301 | BACH1 | 21 | 30671710 | 26 | 0.02 | 0.03 | 0.11 | |
| cg26635603 | BACH1 | 21 | 30671891 | 153 | 0.05 | -0.01 | 0.84 | |
| cg03732014 | BACH1 | 21 | 30676184 | 1375 | 0.49 | -0.02 | 0.50 | |
| cg07083481 | BACH1 | 21 | 30676570 | 989 | 0.92 | 0.01 | 0.82 | |
| cg22076474 | BACH1 | 21 | 30677172 | 387 | 0.39 | -0.07 | 0.18 | |
| cg23530064 | BACH1 | 21 | 30713266 | 35705 | 0.82 | -0.12 | 0.08 | |
| cg19325435 | ZNF280B | 22 | 22839645 | 4077 | 0.90 | -0.08 | 0.10 | |
| cg04971769 | ZNF280B | 22 | 22843648 | 74 | 0.74 | 0.02 | 0.63 | |
| cg24117657 | ZNF280B | 22 | 22859162 | 4342 | 0.92 | 0.00 | 0.99 | |
| cg00127150 | ZNF280B | 22 | 22862144 | 1360 | 0.77 | -0.02 | 0.52 | |
| cg16835233 | ZNF280B | 22 | 22862479 | 1025 | 0.15 | -0.06 | 0.03 | |
| cg13304665 | ZNF280B | 22 | 22862802 | 702 | 0.06 | 0.02 | 0.42 | |
| cg20361154 | ZNF280B | 22 | 22862884 | 620 | 0.02 | 0.01 | 0.75 | |
| cg02440976 | ZNF280B | 22 | 22863054 | 450 | 0.02 | -0.01 | 0.61 | |
| cg15970156 | ZNF280B | 22 | 22863219 | 285 | 0.11 | 0.06 | 0.10 | |
| cg16184943 | ZNF280B | 22 | 22863293 | 211 | 0.28 | 0.02 | 0.52 | |
| cg01223204 | ZNF280B | 22 | 22863655 | 149 | 0.92 | -0.01 | 0.81 | |
| cg10719664 | ZNF280B | 22 | 22863663 | 157 | 0.86 | -0.01 | 0.70 | |
| * coefficient from beta regression adjusted for age, sex, and clinic and cell type | | | | | | | | |

| **Table S5. Effect of adjustment for maternal childhood smoking on the effects of prenatal smoke exposure on DNA methylation in CAMP and Asthma BRIDGE populations**  c)  d)  b)  a) | | | | | | | |
| --- | --- | --- | --- | --- | --- | --- | --- |
|  |  |  | **CAMP** | | **ABRIDGE replication** | | **Evidence of confounding***** |
| **Probe ID** | **Symbol** | **Chr** | **Model 1*** | **Model 2**** | **Model 1*** | **Model 2**** |  |
| cg05697249 | C11orf52 | 11 | 0.09 | 0.09 | 0.06 | 0.06 | no effect |
| cg14724265 | PPEF2 | 4 | 0.17 | 0.16 | 0.11 | 0.13 | no effect/marginal |
| cg09352789 | XPNPEP1 | 10 | -0.06 | -0.04 | -0.07 | -0.06 | Confounding |
| cg25464840 | FRMD4A | 10 | 0.13 | 0.07 | 0.08 | 0.07 | Confounding |
| * Model 1: beta regression modeling effect of IUS adjusted for age, sex, clinic and cell type | | | | | | | |
| **Model 2: beta regression modeling effect of IUS adjusted for age, sex, clinic, cell type and maternal smoking during childhood | | | | | | | |
| ***Confounding defined as >10% change in effect estimate | | | | | | | |
